# High-quality genome (re)assembly using chromosomal contact data

**DOI:** 10.1038/ncomms6695

**Published:** 2014-12-17

**Authors:** Hervé Marie-Nelly, Martial Marbouty, Axel Cournac, Jean-François Flot, Gianni Liti, Dante Poggi Parodi, Sylvie Syan, Nancy Guillén, Antoine Margeot, Christophe Zimmer, Romain Koszul

**Affiliations:** 1 Institut Pasteur, Department of Genomes and Genetics, Groupe Régulation Spatiale des Génomes, 75015 Paris, France; 2 CNRS, UMR 3525, 75015 Paris, France; 3 Institut Pasteur, Unité Imagerie et Modélisation, 75015 Paris, France; 4 CNRS, URA 2582, 75015 Paris, France; 5 Sorbonne Universités, UPMC Univ Paris06, IFD, 4 place Jussieu, 75252 Paris, France; 6 Max Planck Institute for Dynamics and Self-Organization, Group Biological Physics and Evolutionary Dynamics, Bunsenstr. 10, 37073 Göttingen, Germany; 7 Institute for Research on Cancer and Ageing of Nice (IRCAN), CNRS UMR 7284—INSERM U108, Université de Nice Sophia Antipolis, 06107 Nice, France; 8 IFP Energies Nouvelles, 1 et 4 avenue de Bois-Préau, 92852 Rueil-Malmaison, France; 9 Institut Pasteur, Unité Cell Biology of Parasitism, 75015 Paris, France

## Abstract

Closing gaps in draft genome assemblies can be costly and time-consuming, and published genomes are therefore often left ‘unfinished.’ Here we show that genome-wide chromosome conformation capture (3C) data can be used to overcome these limitations, and present a computational approach rooted in polymer physics that determines the most likely genome structure using chromosomal contact data. This algorithm—named GRAAL—generates high-quality assemblies of genomes in which repeated and duplicated regions are accurately represented and offers a direct probabilistic interpretation of the computed structures. We first validated GRAAL on the reference genome of *Saccharomyces cerevisiae*, as well as other yeast isolates, where GRAAL recovered both known and unknown complex chromosomal structural variations. We then applied GRAAL to the finishing of the assembly of *Trichoderma reesei* and obtained a number of contigs congruent with the know karyotype of this species. Finally, we showed that GRAAL can accurately reconstruct human chromosomes from either fragments generated *in silico* or contigs obtained from *de novo* assembly. In all these applications, GRAAL compared favourably to recently published programmes implementing related approaches.

## Abstract (web summary)

The correct assembly of genomes from sequencing data remains a challenge due to difficulties in correctly assigning the location of repeated DNA elements. Here the authors describe GRAAL, an algorithm that utilizes genome-wide chromosome contact data within a probabilistic framework to produce accurate genome assemblies.

## Graphical abstract

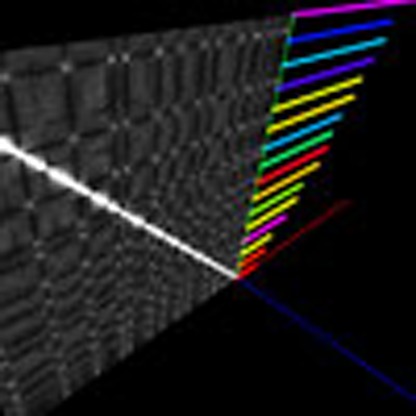

The dropping costs and massive increases in the throughput of next-generation sequencing (NGS) technologies have generated unprecedented amounts of genomic data from various species, strains and tissues. These revolutionary approaches have been accompanied by a number of post-sequencing challenges, notably the finishing of genome assemblies^1^,^2^,^3^. Most NGS technologies currently available generate reads of a few hundreds of base pairs or less. Standard assembly algorithms piece overlapping reads together into larger contiguous sequences (contigs) but usually fail to recover the correct set of chromosomes, leaving many gaps, rearrangements and other errors in the assembly (notably when repeated DNA sequences are present)^4^. Mate-pair or fosmid-end sequencing allows bridging DNA regions separated by at best ~40 kb; however, larger repeated regions are not resolved and remain major sources of chromosome-scale misassemblies^4^. These limitations are not only encountered for large, eukaryotic genomes, but also frequently impair the proper assembly of microbial genomes otherwise well studied for their pathogenic, industrial or evolutionary characteristics.

Scaffolding the contigs into larger structures and eventually closing the gaps between them remains a daunting task that typically requires time-consuming and/or low-throughput, expensive methods. Although novel approaches are constantly and actively sought to address this issue (taking advantage, for instance, of the longer reads offered by new sequencing technologies^5^), only for a few so-called ‘model organisms’ do published assemblies accurately reflect the true linear structure of the genome. Even then, repeats often remain a problem, for instance in regions exhibiting high structural polymorphisms between individuals. In addition, current assembly methods do not provide a framework to assess objectively the reliability of the reconstructed genome sequences. Thus, innovative approaches are needed to exploit fully and extend the power of NGS^6^,^7^,^8^,^9^.

A promising alternative approach was recently pursued by two studies that used Hi-C, a genome-wide application of chromosome conformation capture (3C)^10^,^11^ characterized by an enrichment step, to improve the scaffolding of the human genome^12^,^13^. 3C is a biochemical assay that measures the contact frequencies between pairs of DNA segments in a genome, providing a powerful way to study its three-dimensional (3D) organization^10^,^11^,^14^,^15^. In standard 3C studies, an experimental protocol involving DNA crosslinking, digestion using restriction enzymes, dilution and religation is used to generate a library of DNA fragments that reflects the physical contacts of the DNA molecule(s) within the cell^16^. In its genome-wide derivative Hi-C, the 3C library is enriched in religated products using biotinylation and paired-end sequenced, then a contact frequency matrix is built by mapping the detected religation events along pre-assembled DNA scaffolds (chains of contigs)^11^,^14^,^15^. When these scaffolds reflect the actual chromosomes, that is, when the genome has been correctly assembled, the contact matrix invariably exhibits a strong and broad diagonal reflecting the frequent contacts between adjacent DNA regions within chromosomes. This characteristic feature results from the physical nature of chromosomes, which are semiflexible polymer chains that frequently loop on themselves over small genomic distances^11^,^17^. For genomes with multiple chromosomes, another canonical feature is the presence of prominent blocks along the diagonal indicating that contacts between distinct regions of the same chromosome are generally more frequent than contacts between different chromosomes. However, if the reference genome of the studied organisms has been incompletely or incorrectly assembled, the matrix obtained by mapping the ligation products on the scaffolds or contigs exhibits several incongruities such as contact-enriched blocks located away from the diagonal (Fig. 1a, lower middle and right panels). In addition, when duplicated regions are fused in the reference genome assembly, they appear as a single contig or segment presenting two times more contacts with the rest of the genome than ordinary regions do (because each duplicated copy of a region makes contacts with the rest of the genome, and because all of these contacts are attributed to a single region). Similarly, tale-telling signatures of structural variations between the genome used in 3C/Hi-C experiments and reference sequences can be detected (for instance in cancer cells^18^). These observations suggest that reordering the genome fragments for which incongruent contact signals are detected so that high-frequency contacts are grouped along the diagonal of the matrix may allow assembling the fragments into larger scaffolds.

Two algorithms, called Lachesis^12^ and dnaTri^13^, have used this principle to improve the scaffolding of the human genome. Both apply a two-step procedure: first, Hi-C data are used to cluster contigs into groups sharing high contact frequencies among each other and therefore likely to belong to the same chromosome. Second, the contigs within each group are reordered relative to each other such that contiguous contigs have high contact frequencies, thereby generating chromosomal scaffolds. The final scaffolding obtained by these procedures is likely to reflect the true linear structure of single chromosomes.

These two studies have demonstrated that 3C contact data can be used for improving genome assembly. However, despite these promising advances there remain several important limitations. First, the proposed methods do not account for duplications: notably, repeated regions that were fused during the sequence assembly cannot be resolved using these approaches. Second, an error in the initial clustering step cannot be corrected during scaffolding, making the final genome assembly quality strongly dependent on the clustering accuracy. Third, each algorithm has specific limitations of its own: dnaTri does not attempt to orient the contigs in the scaffolding, whereas Lachesis requires several genome-specific parameters to be specified beforehand, including the exact number of chromosomes^12^, which limits its application to genomes that are already well characterized. Fourth, both methods propose a single genome assembly result, irrespectively of the quality and potential ambiguity of the input 3C data and without providing a global (Lachesis) or stable (dnaTri) probabilistic information about its reliability. The limitations of these algorithms are readily apparent in the validation experiments reported in these two studies, which contained significant imperfections (such as fusions of distinct chromosomes and inversions of up to ~100-Mbp segments by Lachesis^12^).

Starting from similar considerations about the usefulness of genomic 3C data for scaffolding contigs and improving genome assembly, we have developed Genome (Re)Assembly Assessing Likelihood (GRAAL), an independent and powerful new computational approach that largely overcomes the limitations of these first methods. In the following, we first describe the principle of our approach, then demonstrate its ability to (re)assemble accurately known yeast genomes and to identify correctly simple and complex structural rearrangements. Moreover, we apply our method to finishing the assembly of a draft genome and demonstrate its promising application to human chromosomes. We also provide comparisons of our approach to the previously proposed methods^12^,^13^.

## Results

### Principles of the 3C-based genome assembly algorithm GRAAL

**Figure 1:**
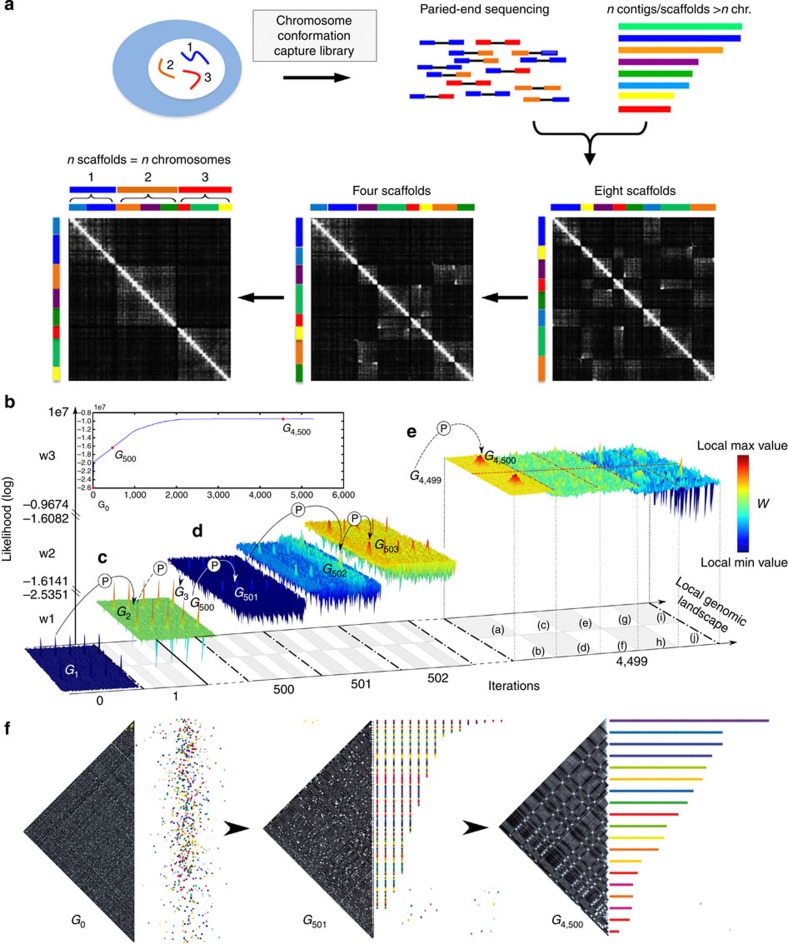
Principle of genomic assembly from chromosomal contact data using GRAAL. (**a**) Upper panels, from left to right: a fictitious genome comprising three chromosomes is processed into a genomic Hi-C library and then paired-end-sequenced. The fictitious genome is not fully assembled, but remains split into eight contigs or scaffolds. To resolve the genome structure, the reads from the Hi-C library are mapped on these contigs, allowing the construction of an initial contact matrix. Lower panels, from right to left: the presence of off-diagonal blocks in this initial contact matrix reflects the imperfections in the original assembly. GRAAL iteratively modifies the set of contigs in order to remove progressively these features and to increase the likelihood of the genome structure given the Hi–C data. In the final steps, the off-diagonal blocks disappear and genome structures that better reflect the 3D contact data are recovered. (**b**) Detailed visualization of the sampling algorithm implemented in GRAAL at three different stages: (**c**) initialization (iterations 0 and 1); (**d**) rapid increase in likelihood (iterations 500–502); (**e**) stabilization and fine-tuning of the structure (iteration 4,500). Because of the huge jumps in likelihood space performed by GRAAL, different scales are used for each of the three windows represented in **c**–**e**. The likelihoods on the *z* axis are represented using the same colour scale for windows w1, w2 and w3 (right panel). Hi-C reads are aligned on a reference genome *G* and the algorithm is initialized with *G*_0_, the set of contigs obtained by splitting *G* into bins of two or more restriction fragments (as determined by the user). At each iteration, a bin is picked at random. This bin is used to explore the local genomic landscape of structural variations around the current genomic structure, whose distribution is represented along the *x* and *y* axes. The planes occupied by the different structural variants are detailed in **e**: single insertion (a, b), insertion and split of contigs (c–f) and translocations (g–j). On the basis of the likelihoods computed for these structures (*z* axis), the sampling algorithm selects the next genomic structure, and a new set of nuisance parameters is sampled (white circles with the letter P, see methods). The algorithm reaches an equilibrium after ~3,000 iterations, which corresponds to the target distribution of optimum genome structures as displayed in **b**. (**f**) Real-time visualization of both the new scaffolds (left) and the corresponding contact maps (right) allows visual monitoring of the progress of the assembly (see Supplementary Movie 1).

We developed GRAAL, an algorithm that iteratively applies virtual rearrangements—or ‘structural variations’ (see below)—to an initial set of DNA fragments, in such a way as to generate one-dimensional (1D) genome structures that are consistent with the 3D contact data (Fig. 1). The method is based on a formulation of the probability of a proposed genome structure as a function of chromosome contact data and prior (data-independent) assumptions relating the expected contact frequencies to the genome structure. These assumptions take advantage of the fact that predicted and observed intrachromosomal contact frequencies are strongly related to the genomic distance between loci, typically following an approximate power law relation and exhibiting a plateau for large genomic distance (at which the frequencies of intrachromosomal and interchromosomal contacts become comparable)^11^,^15^,^19^,^20^.

The set of DNA fragments used for initializing the algorithm is generated by splitting contigs (either generated *de novo* by assembling reads (see below), or already scaffolded) or a reference genome into bins of restriction fragments (RFs). The minimum number of RFs within a bin so that it can be oriented is two. We have implemented a sampling algorithm to determine the probability density of the genome structures that can be obtained by rearranging these bins (see Methods and Supplementary Information). Briefly, at each iteration the GRAAL algorithm draws a genomic bin randomly without replacement, then picks a number *m* of partner bins (defined by the user) with probabilities that depend on the measured contact frequencies. GRAAL then considers 14 different types of virtual ‘mutations’ involving these bins and mimicking biological rearrangements such as inversions, insertions, deletions and, importantly, duplications. These mutations define a set of 14 × *m* candidate genomes whose likelihoods are computed using the aforementioned model. One of the structures with the highest likelihoods is then retained for the next iteration (Methods and Supplementary Information). Once all the bins have been visited, a new cycle starts that reprocesses the entire set of genomic bins starting from the most likely structure from the former cycle. Overall, thousands of iterations are applied by GRAAL and the position of each genomic bin is revisited several times depending on the number of cycles specified by the user.

GRAAL significantly differs from previous methods^12^,^13^ in at least three key aspects: (i) as our algorithm independently and repeatedly examines the position of every single bin (consisting of two or more RFs) in the genome rather than relying blindly on pre-assembled contigs, GRAAL is able to correct assembly and scaffolding errors and to characterize very small structural variations (the resolution limit being the size of the bin); (ii) GRAAL is able to identify and position duplicated regions, which are the key obstacles to standard assembly programmes; and (iii) GRAAL’s sampling approach, which determines not just one structure but a family of likely structures and their associate probabilities, provides an objective measure of the likelihood of the genome assemblies given the Hi-C data set at hand. These advantages are illustrated by the results and comparisons detailed below.

### Validation of GRAAL on budding yeast contact data

**Figure 2:**
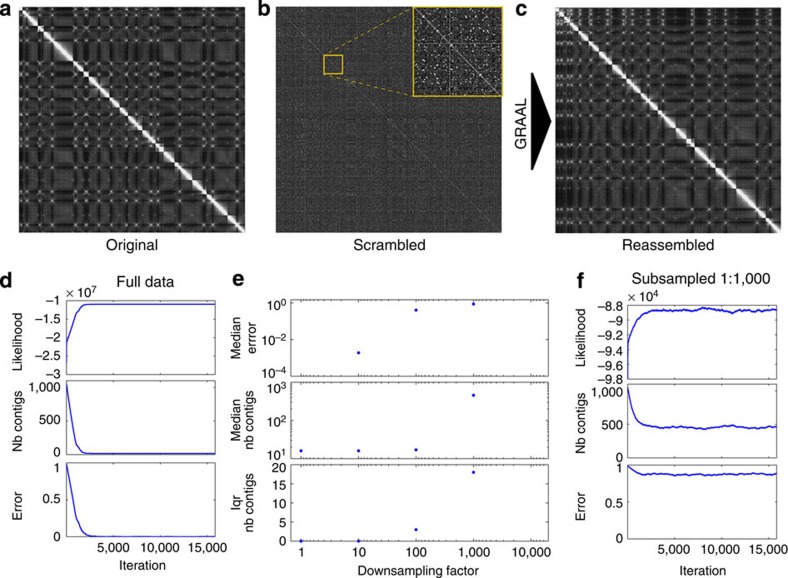
Quantitative validation of genome assembly by GRAAL on the budding yeast genome. (**a**) *Saccharomyces cerevisiae* contact matrix obtained by mapping 3C-seq reads on the reference genome. (**b**) Contact matrix from the same contact data after random permutation of the 1,086 contigs. The inset shows a close-up on a block along the diagonal. (**c**) Contact matrix after reassembling the contigs using GRAAL. (**d**) Likelihood, number of contigs and assembly error plotted as a function of the number of iterations when GRAAL was run on the full contact data set. (**e**) Effect of randomly downsampling the contact data set. The plots show the median assembly error, median and interquartile range of the number of contigs as a function of the downsampling factor, ranging from 1 (original data) to 1,000 (0.1% of the reads chosen randomly). (**f**) Same as **d**, but with the contact data downsampled by a factor 1,000.

As a validation of our method, we first applied it to the budding yeast *Saccharomyces cerevisiae* strain BY4741 (the linear genome structure of which had been fully characterized^21^). We used 3C-seq, agenome-wide derivative of 3C that does not involve an enrichment step (Methods), to generate a genome-wide contact matrix (3,240 × 3,240) featuring 10,497,600 *trans*- and *cis*-chromosomal contacts (Fig. 2a; Methods). The genome was then split into 1,086 bins of ~11 kb each that were randomly reordered, thereby scrambling the original matrix (Fig. 2b). GRAAL was initialized with this set of bins and the algorithm was allowed to run for over 15,000 iterations (Fig. 2c and Supplementary Information). During the first 2,000 iterations, the likelihood increased consistently while the number of contigs progressively decreased and the difference between the reconstructed and true linear structure of the genome diminished rapidly; after roughly 4,000 iterations, the likelihood and number of contigs essentially stabilized and the amount of errors in the resulting assembly (defined as in the Supplementary Information) remained close to zero (Fig. 2d). Although the algorithm still sampled the structure space, oscillations of structural parameters such as the number of contigs were very rare. The interquartile range of the number of contigs after 5,000 iterations, iqr(*N*_contigs_), hereafter used as a measure of structure dispersion (Supplementary Information), was zero, indicating that the likelihood peaked sharply around a unique structure (Fig. 2e). The median number of contigs was 16, corresponding to the true number of chromosomes, and the median reconstruction error (taking into account both the orientation and the relative order of the bins) after stabilization was zero, indicating an excellent agreement between the reconstructed and the true genome structure (Fig. 2c,e). By contrast, the clustering algorithm of dnaTri failed to recover the expected number of chromosome clusters and predicted only 2 of them. Lachesis, when instructed to seek 16 chromosomes, pooled together bins belonging to different chromosomes (for example, small chromosomes 1, 3 and 6) while splitting others (Supplementary Fig. 1). Overall, ~15% of the bins were improperly clustered by Lachesis, although 95% of them remained positioned next to their correct neighbours when compared with the reference genome, reflecting the fact that inappropriate clustering concerned mostly stretches of contiguous bins. The results from both dnaTri and Lachesis stand in sharp contrast with GRAAL, which, using the same data, was able to reconstruct the 16 chromosomes with absolutely no error and did not require prior knowledge of the number of chromosomes.

In order to highlight both the probabilistic nature and the robustness of the reconstructions obtained using GRAAL, we ran our algorithm on random subsets of our contact data (Fig. 2e). As expected, downsampling the contact data by including only a fraction of the reads led to a progressive deterioration of the quality of the reconstruction; however, with a tenfold downsampling the median error was less than 2 × 10^−3^ (Fig. 2e, top), indicating that the genome (re)assembly remained very good even when only ~1.5 million reads were included. The assembly quality only broke down when downsampling by a factor of >100, in which case the structural error exceeded 40%. For such sparse data, the algorithm did not converge towards a single structure: instead, it kept exploring a much wider structural space, resulting in strong oscillations in likelihood and contig numbers (for example, iqr(*N*_contigs_)>15 for 1/1,000th downsampling; Fig. 2e,f; Supplementary Fig. 2). In addition to highlighting the known importance of sequencing depth for accurate genome assembly^13^, the fact that GRAAL still yielded excellent results despite a tenfold downsampling of the data underlines the robustness of our approach.

Another important property of GRAAL is illustrated by the above analysis: by construction, optimization approaches produce a single solution, regardless of the quality of the experimental data. For poor data, this structure may be widely incorrect, but optimization methods typically provide little clues about the associated uncertainty. By contrast, GRAAL, being based on a probabilistic sampling approach, generates a distribution of structures with their associated likelihoods. This effectively allows determining whether or not unique structures can reliably be inferred from the data, using measures of the structure dispersion such as iqr(*N*_contigs_; Fig. 2e, bottom). Importantly, these measures do not require *a priori* knowledge of a reference genome sequence, making it possible to evaluate the assembly uncertainties for new, unknown genomes.

### GRAAL identifies chromosomal rearrangements

**Figure 3:**
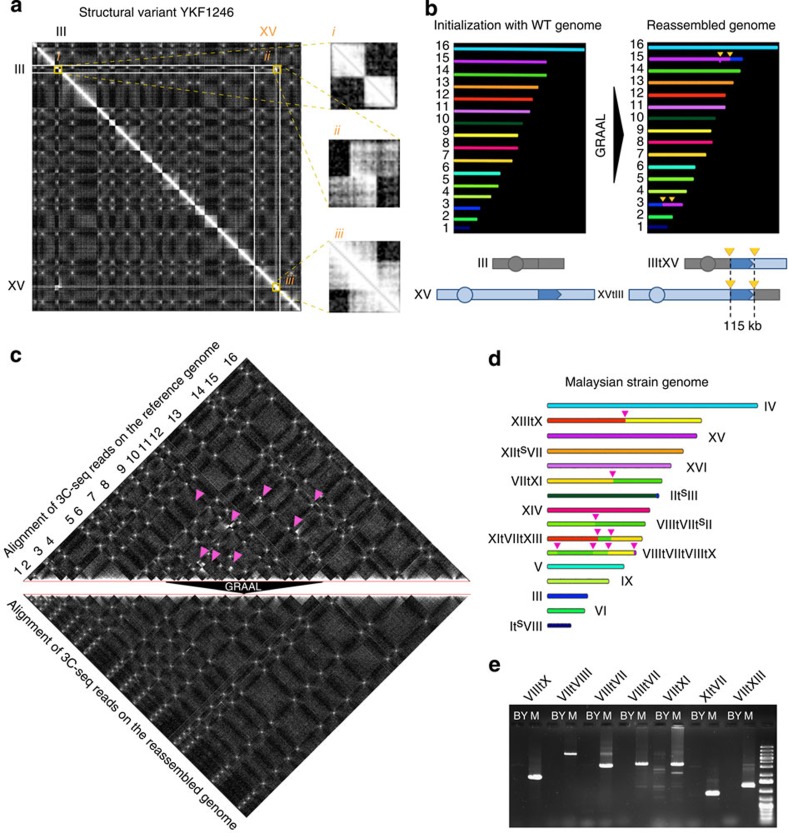
Identification of structural genome rearrangements using GRAAL. (**a**) *S. cerevisiae* strain YKF1246 contact matrix obtained by mapping 3C-seq data to the reference wild-type genome revealed intra- (i, iii) and interchromosomal (ii) incongruities affecting chromosomes 3 and 15. (**b**) GRAAL initialized with the reference genome and the YKF1246 contact matrix generated a reassembled genome carrying a ~100-kb duplication and a translocation between chromosomes 3 and 15 (yellow arrows), as expected^22^. (**c**) Malaysian yeast strain UWOPS03-461.4 contact matrix obtained by mapping 3C-seq data to the reference wild-type *S. cerevisiae* genome (top panel) revealed interchromosomal incongruities (pink arrows). GRAAL reconstructed a genome structure exempt of these features (bottom panel). (**d**) Structure of the Malaysian yeast strain, with pink arrows representing the breakpoints of the translocations readily visible in the contact matrix of **c**, top. (**e**) Experimental PCR validation of the breakpoints identified by GRAAL. BY: reference strain (BY4741). M: Malaysian strain.

Next, we proceeded to test the algorithm’s ability to identify chromosomal rearrangements, an important feature for studying genome dynamics. We generated contact data for a yeast strain (YKF1246) known to carry two structural chromosomal rearrangements compared with its parental strain: a 115-kb segmental duplication from chromosome 15 on chromosome 3, associated with a translocation of the extremities of these two chromosome arms^22^. The contact matrix was built by mapping the contact reads on the parental genome, revealing clear intra- and interchromosomal incongruities (Fig. 3a, i–iii). Starting from this initialization, GRAAL converged on a nearly unique new genome structure (iqr(*N*_contigs_)=1) that faithfully recapitulated both rearrangements with an accuracy of 2–10 kb (Fig. 3b; Supplementary Fig. 3 and Methods). This ability to identify and correctly assign duplicated regions, without false positives (such as artificial fusions of small chromosomes or large inversions), illustrates a unique feature of GRAAL compared with previous methods^12^,^13^.

To test GRAAL on a more complex set of genomic rearrangements, we turned to a natural Malaysian isolate of *S. cerevisiae*, whose genome was likely to present extensive structural variations compared with the reference genome^23^. Indeed, the contact matrix obtained after mapping 3C-seq reads to the reference genome sequence revealed obvious incongruities (Fig. 3c, top, pink arrows). To determine the structure of the Malaysian strain genome, GRAAL was initialized with the *S. cerevisiae* reference genome together with the Malaysian strain genome contact data. The algorithm then converged towards a single genome structure (iqr(*N*_contigs_)=0). The genome obtained after convergence (Fig. 3c, bottom; see also Supplementary Data 1 and 3) displayed eight chromosomal translocations (resulting from four reciprocal translocations) compared with the reference *S. cerevisiae* genome, as well as four smaller subtelomeric translocations and a few intrachromosomal inversions of small DNA regions that were virtually undetectable by visual inspection of the contact matrix (Fig. 3c,d). We verified the identified breakpoints using PCR amplification and sequencing and confirmed all large intrachromosomal translocations (Fig. 3e). These translocations were also confirmed using PacBio sequencing (GL, personal communication). Small translocations in subtelomeric regions were not amplifiable because of the repeated nature of these sequences and remained invisible using the PacBio technique; however, the subtelomeric regions of yeast chromosomes have been known to be dynamic and recombigenic for a long time, and similar observations have been frequently made among natural variants^24^,^25^. As often observed in natural and laboratory strains, all intrachromosomal breakpoint regions were associated with transposable element sequences presenting long stretches of identity^22^,^26^.

The ability of GRAAL to identify and position accurately large and small chromosomal rearrangements, including duplications, highlights its value for studies involving comparison of the genome structure of closely related strains, as well as for investigating the genome structure of cancerous cell lines.

### Finishing and curation of the *Trichoderma reesei* genome assembly

**Figure 4:**
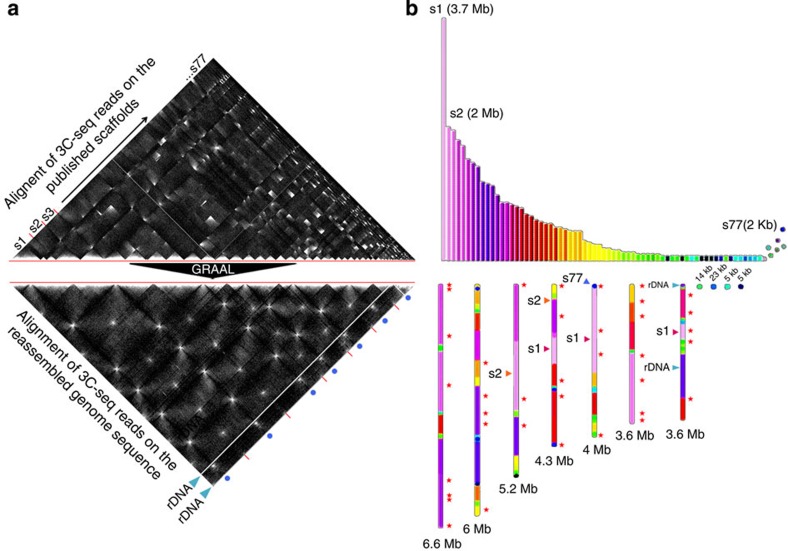
Finishing the *Trichoderma reesei* genome assembly using GRAAL. (**a**) Top panel: *T. reesei* contact matrix obtained by mapping 3C-seq data of strain QM6a against the 77 scaffolds (s1, s2, … s77) of its published genome^27^. Bottom panel: matrix obtained by GRAAL after 31,920 iterations. Red bars indicate the boundaries of the seven chromosome scaffolds; centromere positions are represented by blue dots. Enhanced interactions are apparent between telomeres as well as between centromeres. Blue arrowheads indicate the positions of the ribosomal DNA clusters. (**b**) Top panel: schematic representation of the 77 scaffolds assembled previously^27^. Bottom panel: genome structure proposed by GRAAL after 31,920 iterations. Scaffolds s1, s2 and s77 are tracked from the original assembly into the new structure.

To test GRAAL’s potential for finishing draft genomes, we turned to the filamentous fungus *T. reesei*. This fungus is used all around the world for producing cellulases and is essential for the emerging biomass-to-biofuel industry. Traditional assembly methods for the genome of the QM6a reference strain had yielded 77 scaffolds^27^, whereas electrophoretic karyotyping experiments performed in the 90s had identified only seven chromosomes^28^,^29^. Large variations in chromosome size had been observed between low- and high-producer strains, suggesting a role of chromosome structure in strain performance. To finish the assembly and reveal the complete genome structure of QM6a, we mapped newly acquired 3C-seq data from QM6a on the original published set of 77 scaffolds and obtained a matrix that exhibited a mosaic-like pattern, strongly suggesting that the actual number of chromosomes was much smaller than the number of scaffolds (Fig. 4a). GRAAL dramatically reduced the number of contigs and again essentially converged on a unique (iqr(*N*_contigs_)=0), much improved structure consisting of only seven well-defined super-scaffolds ranging in size from 3.6 to 6.6 Mb and comprising 99.8% of the 33.3-Mb genome, in accordance with the known karyotype (Fig. 4b; Supplementary Movie 1 and Supplementary Data 2 and 4). Only eight small regions remained unassembled, representing a total of 63 kb (<0.2% of the initial assembly), either because they contained no restriction sites for the chosen enzyme or because contact data were insufficient to position them unambiguously. Future 3C-seq or Hi-C experiments using a different enzyme and/or increased sequencing depth will most probably be required to position these small regions into the structure.

The contact matrix obtained after assembly displayed no incongruities and contained features strikingly similar to the budding yeast genome, notably a clustering of centromeric sequences indicative of a Rabl-like chromosome configuration^14^,^30^,^31^. The fact that centromere clustering was not a prior assumption of the simple physical model underlying GRAAL underscores the robustness of our approach with respect to peculiarities of the nuclear organization in different organisms. Other structural differences between the reference genome and the sequenced strain include a 25-kb bin containing ribosomal DNA sequences, found by GRAAL to be duplicated four times on the smallest chromosome (blue arrows in Fig. 4b), and several scaffolds from the original assembly found to be split in two or three smaller fragments dispatched along the seven true chromosomes (s1 and s2 in Fig. 4b). The same structural variations were all observed in the genome of another derivative of the QM6a strain, thereby confirming this finding (data not shown). These results underscore GRAAL’s ability to identify repeated DNA sequences and to correct the errors inherent to standard assembly methods. Analysis of the finalized *T. reesei* genome further revealed that 13 out of 35 clusters of carbohydrate genes (stars in Fig. 4b), which encode the cocktail of biomass-degrading enzymes of this species, are positioned in subtelomeric regions. This is reminiscent of the organization described for the MAL and SUC gene families involved in sugar utilization in budding yeast^32^, a finding that opens new perspectives on the industrial evolution of this strain’s genome and its derivatives.

### Reassembly of human chromosomes from virtual and *de novo* contigs

Finally, we wanted to assess how GRAAL would perform on larger chromosomes/genomes. Because of the computational cost of the approach, we limited ourselves to reassembling a subset of human virtual contigs generated by slicing small and long chromosomes *in silico*, and to assembling *de novo* contigs of chromosome 14 generated by the ALLPATHS-LG^33^ program using sequencing libraries downloaded from the GAGE competition website^34^.

**Figure 5:**
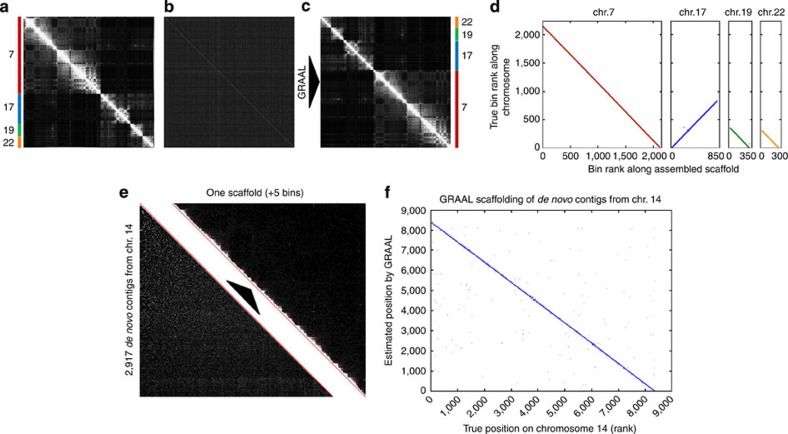
High-resolution reassembly human contigs using GRAAL. (**a**) Contact matrix obtained by mapping published Hi-C reads on chromosomes 7, 17, 19 and 22 of the human genome assembly hg19. (**b**) Contact matrix from the same Hi-C data after dividing the reference sequence into 3,607 virtual contigs and permutating them randomly. (**c**) Contact matrix after reassembly of the virtual contigs using GRAAL. (**d**) Comparison between the reference sequence of the four chromosomes and the sequence of the four scaffolds recovered from the GRAAL assembly. (**e**) GRAAL assembly of 2,917 *de novo* contigs from chromosome 14 (bottom left contact map) into one large scaffold (upper right contact map). (**f**) Comparison of the order of the bins from chromosome 14 as assembled by GRAAL with the order expected from the reference sequence.

Chromosomes 7, 17, 19 and 22 of the reference hg19 genome were split into 3,607 bins of an average size of 100 kb (median size 76 kb; Fig. 5a,b). These bins were used to initialize GRAAL and the programme was run for 10 cycles (that is, 36,070 iterations). At the end of the process, we observed that the bins were distributed within four scaffolds, each corresponding to one of the original chromosomes (Fig. 5c). GRAAL did not exhibit a tendency to fuse small chromosomes together (neither for *S. cerevisiae* nor for *Homo sapiens*), in contrast with Lachesis when applied to both yeast and human data. In addition, 100% of the bins were assigned by GRAAL to the correct chromosome, and overall 97.8% of the bins were accurately ordered (Fig. 5d).

We then applied GRAAL to the assembly of *de novo* contigs of chromosome 14 (ref. 13). From the set of 4,722 initial *de novo* contigs assembled using ALLPATHS-LG (N50=21 kb)^34^, 2,917 contigs were retained after a filtration step and split into 8,382 bins comprising at least three RFs; Supplementary Data 5 and 6). This set of bin was used to initialize GRAAL. After 28 cycles, we observed that 8,377 bins (99.94%) had been reordered into a unique scaffold (Fig. 5e). This scaffold was then aligned against the reference sequence of chromosome 14, revealing no important structural incongruence (mean and median rank errors: 3.9 and 1, respectively; Fig. 5f) and an average positioning error along the chromosome 14 adapted sequence estimated at 23.9 kb (median 149 kb). This illustrates the ability of GRAAL to scaffold contigs generated *de novo*, without the need for traditional mate-pair libraries.

Overall, our results highlight the broad scope of applications of GRAAL, which yielded highly accurate reconstruction of genomes of very different sizes such as those of yeast and human without prior knowledge of their chromosome numbers or of contact frequencies (See extended Methods).

## Discussion

Most genomes sequenced so far are only partially assembled. For example, the genome of the mosquito *Aedes aegypti*, the dengue vector, is currently available only as a set of 4,757 contigs^35^. This severely complicates comparative genomic studies, notably when trying to understand genome evolution. Incomplete genomes also impair quantitative trait locus analyses, making genetic mapping very challenging^36^. Moreover, subtelomeric regions, rich in accessory genes and harbouring ~25% of quantitative trait loci in *S. cerevisiae*, are prone to complex genomic rearrangements and therefore hard to sequence and assemble^25^. This led us to develop GRAAL, a powerful and elegant method that uses the 3D contacts of the DNA molecule within the cell to efficiently finalize genome assemblies. In contrast to standard assembly techniques, our method accurately recovers the actual number and DNA content of chromosomes and does not require expensive and lengthy follow-up experiments.

We have shown that GRAAL can correctly recover the structure of the ~12-Mbp reference budding yeast genome from ~1.5 million 3D contact reads and that it correctly identifies complex structural rearrangements in other yeast strains. We have applied our method to the genome of *T. reesei*, a fungus of industrial importance for biomass degradation and biofuel synthesis^37^. The complete genome structure obtained here will enable the analysis of genetic traits important for massive production of the enzymes involved in these processes and will allow the study of the 3D organization of its genome. We expect our method to dramatically increase the power of similar studies of genome structure and function in various other organisms.

GRAAL can also identify chromosomal rearrangements (including duplications) with high accuracy. Because the position of each bin in the structure is only dependent on the contact data, the likelihood of each structural variation identified can be objectively calculated. No large artefactual chromosome rearrangements were detected in the analyses above, and we believe that GRAAL should be widely applicable to the detection of variations in genome structure, for instance when investigating cancer cells or in experimental evolutionary studies.

We compared GRAAL with two recently described methods that also aim to improve genome scaffolding through the exploitation of chromosome contact data^12^,^13^. GRAAL differs from these two approaches in several important aspects (Supplementary Table 1, Supplementary Fig. 4). First, our algorithm requires only the 3D contact data and a set of contigs as input; no other parameters or manual adjustments are needed, and GRAAL can be applied to any species. Second, unlike these other methods our algorithm identifies and positions correctly duplications in these genomes, which is a major issue for standard genome assembly methods. Third, GRAAL is also unique in allowing to correct misassemblies and to detect chromosome rearrangements, with a genomic resolution only determined by the binning of the initialization contigs. Fourth, as opposed to the sequential clustering and reordering steps used in both Lachesis and dnaTri, GRAAL iterative reshuffles the entire genome structure, using a variety of biologically inspired virtual rearrangements. This greater flexibility may help to explain why GRAAL, originally designed to reassemble yeast genomes, also achieved a very high scaffolding accuracy on human chromosomes without any specific adaptation or assumption, despite the huge difference in genome size and nuclear architecture between yeast and human. Finally, GRAAL provides an explicit likelihood score for each computed genome structure, enabling an objective assessment of the quality of the reconstruction it proposes: this feature is likely to be very valuable for quantitative downstream analyses, for example, of genome structure evolution.

Although GRAAL considerably improves the blind reassembly of complex genomes, it remains perfectible. The accuracy of the final assembly depends not only on the sequencing coverage (see Fig. 2e) but also on the size of the genomic bins used in the algorithm, which is limited by the available memory and computing power. To benefit fully from the available resources, GRAAL runs multiple threads on both central processing units and graphical processing units^38^. Future increases in computing performances will therefore enable GRAAL to process smaller bins and/or larger genomes, making high-resolution (re)assemblies of *de novo* contigs of large genomes accessible. So far, small genomes (<200 Mb) can be processed within a few hours at a genomic resolution of ~11 kb. The four human chromosomes were processed in 12 h on a laptop at a resolution of ~95 kb (Laptop Asus RoG with NVIDIA GTX780M).

Finally, the basic genomic unit remains the RF, and the precise junctions between bins that were not initially neighbours are not resolved. Convergent walking along the chromosome, or longer reads, may ascertain some of these regions and future versions of GRAAL could incorporate such improvements. Another planned improvement is the estimation of the size of the gaps between contigs^13^. Overall, we believe that GRAAL provides a powerful and robust new solution for assembling genomes and identifying chromosomal rearrangements, with far-reaching implications for genomic, metagenomic and genetic research.

## Methods

### Construction of yeast 3C libraries

3C libraries of the *S. cerevisiae* strains were generated from log-phase cells growing in YPD medium, using a frequent cutter (*Dpn*II) and a protocol inspired by several 3C-based protocols^10^,^11^,^15^,^39^. The BY4741 strain^21^, YKF1246 strain^22^ and UWOPS03-461.4 Malaysian strain^40^ were grown overnight in 50 ml YPD at 30 °C. The next day, cultures were diluted in 300 ml YPD to 0.2 × 10^7^ cells ml^−1^ and incubated at 30 °C with agitation until reaching 1 × 10^7^ cells ml^−1^. Cells were then processed as described previously^31^ and the resulting 3C library was quantified on a gel using the programme QuantityOne (Bio-Rad).

### Construction of *T. reesei* 3C libraries

The *T. reesei* strain used in this study was QM6a (ATCC 13631)^27^. Frozen spores were used to inoculate five 1.2 L Roux culture flasks each containing 200 ml of potato-dextrose medium. After incubation at 30 °C for 4 days, the content of the flasks was filtrated and the mycelium transferred into 200 ml of KPAm buffer (0.6 M (NH_4_)_2_SO_4_, 25 mM KH_2_PO_4_, pH 5.8). After incubation at 37 °C for 30 min in an orbital shaker at 150 r.p.m., the solution was filtrated and the mycelium recovered in a Schott bottle containing 100 ml of KPAm buffer with 30 mg ml^−1^ of lytic enzymes (Glucanex, Novozymes). After 2 h 30 min at 37 °C in an orbital shaker (150 r.p.m.), the solution was filtrated through a fritted glass Büchner funnel n°1 and the protoplasts were collected by centrifugation (4,000 *g* at 4 °C for 5 min). The protoplast pellet was resuspended in 25 ml CTS10 solution (0.4 M sucrose, 0.1 M Tris HCl pH 7.5, 10 mM CaCl_2_), centrifuged (4,000 *g* at 4 °C for 5 min) and the supernatant was discarded. The pellet was then dissolved in 10 ml CTS10 solution and centrifuged (4,000 *g* at 4 °C for 5 min). Finally, the protoplast pellet was resuspended in 5 ml of CTS50 solution (0.4 M sucrose, 50 mM CaCl_2_, 0.1 M Tris HCl pH 7.5) and the concentration adjusted to 3 × 10^8^ cells ml^−1^. The protoplast solution (4 ml) was treated with fresh formaldehyde (Sigma Aldrich—36.5–38% in H_2_O; 2% final concentration) for 20 min at room temperature (RT). The remaining formaldehyde was quenched with glycine (0.25 M final concentration) for 5 min at RT and 15 min at 4 °C. The fixed protoplasts were then collected by centrifugation and stored at −80 °C until use. 3C libraries were built as described previously^31^ and the resulting 3C library was quantified on a gel using the programme QuantityOne (Bio-Rad).

### Processing 3C libraries into 3C-seq libraries ready for sequencing

Aliquots of 5 μg of each 3C library were dissolved in water to a final volume of 130 μl and then sheared using a Covaris S220 instrument (duty cycle 5, intensity, 5,200 cycles per burst, four cycles of 60 s each). The sheared DNA was subsequently purified on a QIAquick column and then processed using the Illumina Paired-End DNA Sample Prep Kit (PE-930-1001). DNA was ligated to modified Illumina PE adapters (see Supplementary Table 2) for 3 h at RT in a final volume of 30 μl (20 μl of DNA (~8 μg), 3 μl of ligation buffer 10 × (NEB), 3 μl of T4 DNA ligase (400 U μl^−1^; from NEB) and 4 μl of 10 μM adapter solutions). The tubes were then incubated at 65 °C for 20 min. DNA molecules with sizes comprised between 400 and 800 pb were purified using the PippinPrep apparatus (SAGE science) and amplified using Phusion (Finnzymes). The PCR products were purified on Qiagen MinElute columns and sequenced.

### Processing of PE reads and construction of a contact matrix

The raw data from all 3C-seq experiments were processed as follow: first, short reads were mapped on the genomes of *S. cerevisiae* (GCF_000146045.1) and *T. reesei* QM6a (GCA_000167675.2) using bowtie2 in local and very sensitive mode^41^. Only pairs of reads with a mapping quality above 30 were retained, and contact reads mapping on the same fragment were discarded^17^. PCR duplicates were also removed using the six Ns present on each custom-made adapter (see Supplementary Table 2).

### Computational method for genome assembly

An exhaustive description of GRAAL is available in the Supplementary Information file accompanying this article. The Supplementary Table 3 recapitulates all the different initialization parameters and data sets used in the different analyses. The GRAAL program is available as an accompanying file to this article (graal.zip), with a readme file containing instructions on how to use it (Marie-Nelly *et al*.—GRAAL readme.pdf). GRAAL can also be downloaded using the link https://github.com/koszullab/GRAAL and on the laboratory websites of R.K. and C.Z.. Here we provide a short summary of the GRAAL algorithm. Given an experimental chromosomal contact data set *D*, GRAAL seeks to explore the probability distribution *p*(*G*|*D*) of the 1D genome structure(s) *G* consistent with the data. The algorithm is based on a probabilistic approach inspired by earlier work on protein structure determination^42^ and employs Bayes’ rule *p*(*G*|*D*)∝*p*(*D*|*G*)*p*(*G*), where *p*(*G*|*D*) is the posterior, *p*(*D*|*G*) the likelihood and *p*(*G*) is the prior. Assuming that in the absence of data all structures *G* have equal probability (flat prior), this reduces to: *p*(*G*|*D*)∝*p*(*D*|*G*). The calculation of *p*(*D*|*G*) requires a model to quantitatively predict the *cis*- and *trans*-chromosomal contacts for a given *G*. To do so, we assume the *cis*-contact probabilities *P*_c_ to depend on the genomic distance *s* as a power law followed by a plateau: 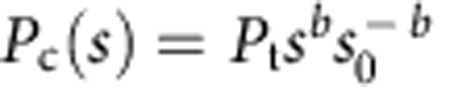 for *s*≤*s*_0_ and *P*_c_(*s*)=*P*_t_ for *s*≥*s*_0_, in accordance with the theoretically predicted and measured behaviour of chromosomes confined in a nucleus^11^,^14^,^20^,^43^. Different values of *b* and *s*_0_ have been reported for different organisms or chromosomes^11^,^14^,^20^,^43^,^44^. We assume that *trans*-contacts occur with uniform probability *P*_t_ per unit genomic length squared. The three so-called nuisance parameters *ξ*=(*b, s*_0_*, P*_t_) are not fixed in advance, but are also estimated by GRAAL (see below) using a flat prior. Finally, we assume that the counts of the measured contact matrix *D* obey a Poisson distribution, that is, that 
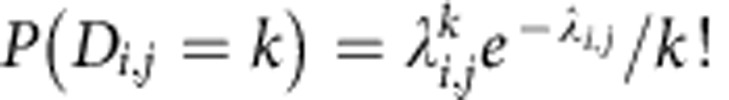
 (*k*ε*N*), where the contact probability *λ*_*i,j*_ for bin *(i, j)* is given by *P*_t_ or *P*_c_ for *trans* or *cis* contacts, respectively. Together, these assumptions specify a probabilistic model *p*(*G*, *ξ|D*) that allows to calculate the likelihood *p*(*D*|*G*, *ξ*) of any genome structure *G* given a Hi-C or 3C-seq data set *D*.

In addition, GRAAL requires a method to sample the space of possible nuisance parameters and genome structures, which is infinite. The nuisance parameters ξ are updated iteratively in alternation with the changes of genome structure by a classic Metropolis algorithm (see Supplementary Fig. 2a,b). In order to generate the genomic structures we implemented a stochastic sampler inspired by the Multiple-Try Metropolis algorithm^45^,^46^, which generates an ordered sequence of genome structures *G*_*t*_, *t*=1, 2….*N*_*t*_ starting from an initial guess *G*_0_. Given a current genome structure *G*_*t*_, a random set of *N* new structures is computed by applying 14 different virtual structural changes including insertions, deletions, duplications, inversions, translocations and transpositions (jumps). For each new candidate structure, GRAAL computes the likelihood using the probabilistic model summarized above, and one of these structures is chosen with a probability determined by its likelihood. To alleviate the computational load, and in contrast to the classical Multiple-Try Metropolis rule^46^, the new genome *G*_*t*+1_ is systematically accepted. As opposed to a uniformly random or deterministic choice of structural variations, this procedure allows for computationally efficient sampling of the structure probability density. Finally, after discarding a burn-in period, the Markov chain samples are used to estimate the joint probability distribution of (*G*, ξ). The GRAAL program, including its source code and a graphical user interface, are freely available for non-commercial purposes.

## Author contributions

H.M.-N. designed and developed the algorithm with support from C.Z. and R.K. M.M. performed the experiments. M.M., N.G. and R.K. conceived the experiments. AC and J.-F.F. provided support with the genomic analyses. G.L. provided the Malaysian yeast strain and confirmed independently the rearrangements identified by GRAAL, whereas D.P.P. and A.M. provided the *T. reesei* cells used in the study. R.K. and C.Z. wrote the paper, with help from J.-F.F., G.L., M.M., H.M.-N. and A.M. C.Z. and R.K. supervised the research; R.K. designed and coordinated the project.

## Additional information

**How to cite this article:** Marie-Nelly, H. *et al*. High-quality genome (re)assembly using chromosomal contact data. *Nat. Commun.* 5:5695 doi: 10.1038/ncomms6695 (2014).

**Accession codes:** Genome-wide data generated in this study have been deposited in the Sequence Read Archive (SRA) under accessions no. SRP049758, SRP049761 and SRP049800.

## Supplementary Material

Supplementary Figures, Tables, Methods, and References Supplementary Figures 1-4, Supplementary Tables 1-3, Supplementary Methods and Supplementary References

Supplementary Movie 1 Iterative reassembly of Trichoderma reesei genome using genomic contact data, showing the selected genome structure on the right and the corresponding contact matrix on the left. The contigs and scaffolds of the genome structure on the right panel are ordered according to their size along the vertical axis, with the longest scaffolds positioned at the top and the smallest ones at the bottom. During the first part of the movie, the original assembly of 76 scaffolds is binned into 1200 elements. The assembly process has not started yet. Once this binning is complete, new genome structures are iteratively tested, one bin at a time (Figure 1F). Arrays of syntenic bins are indicated along the horizontal axis on the right, with the same color code as in the original assembly. At first, very small scaffolds encompassing only a few bins appear, that grow progressively longer. The likelihood and the number of scaffolds of the current structure are indicated in the left panel. When the iterative process stops, the ongoing structure presents 7 large scaffolds covering 99.8% of the original set of 76 scaffolds, and 4 small bins. If allowed to run longer, the algorithm would keep fine-tuning the genome structure by looking for small improvements in the likelihood.

Supplementary Data 1 Most likely genome structure for the Malaysian yeast strain after 447,880 iterations

Supplementary Data 2 Most likely genome structure for the T. reesei strain QM6A after 1,331,920 iterations

Supplementary Data 3 Fasta file of the most likely genome structure of the UWOPS03- 22 461.4 Malaysian yeast strain after 47,880 iterations

Supplementary Data 4 Fasta file of the most likely genome structure of the T. reesei strain 25 QM6A after 31,920 iterations

Supplementary Data 5 List of the 2,917 de novo contigs of chromosome 14 from sequencing 28 libraries downloaded from the GAGE competition website used for initializing GRAAL

Supplementary Data 6 List of the 8,382 bins generated from these 2,917 contigs from 31 Supplementary Data 5
