# Supplementary material for: High-quality genome (re)assembly using chromosomal contact data
Source: Nat Commun. 2014 Dec 17;5:5695. doi: 10.1038/ncomms6695 (PMC4284522; doi:10.1038/ncomms6695)
Supplement: Supplementary Figures, Tables, Methods, and References — Supplementary Figures 1-4, Supplementary Tables 1-3, Supplementary Methods and Supplementary References [file ncomms6695-s1.pdf]

**Supplementary Information for Marie-Nelly et al.**

**Supplementary Data 1. Most likely genome structure for the Malaysian yeast strain after 47,880 iterations**

This file generated by GRAAL recapitulates the correspondence between the genome used for the initialization of GRAAL and the most likely genomic structure recovered at the end of the process. Superscaffolds generated by GRAAL are indicated in the first column, with the corresponding bin from the original genome indicated below each superscaffold under the "init_published_scaffold" label. The index, orientation, and initial coordinates of each bin within the initial genome sequence are also indicated.

**Supplementary Data 2. Most likely genome structure for the *T. reesei* strain QM6A after 31,920 iterations**

This file generated by GRAAL recapitulates the correspondence between the genome used for initializing the algorithm and the most likely genomic structure recovered. Superscaffolds generated by GRAAL are indicated in the first column, with the corresponding "bin" from the original genome indicated below each superscaffold under the "init_published_scaffold" label. The index, orientation, and initial coordinates within the initial genome sequence are also indicated.

**Supplementary Data 3. Fasta file of the most likely genome structure of the UWOPS03-461.4 Malaysian yeast strain after 47,880 iterations**

**Supplementary Data 4. Fasta file of the most likely genome structure of the *T. reesei* strain QM6A after 31,920 iterations**

**Supplementary Data 5.** List of the 2,917 *de novo* contigs of chromosome 14 from sequencing libraries downloaded from the GAGE competition website used for initializing GRAAL.

**Supplementary Data 6.** List of the 8,382 bins generated from these 2,917 contigs from Supplementary Data 5.

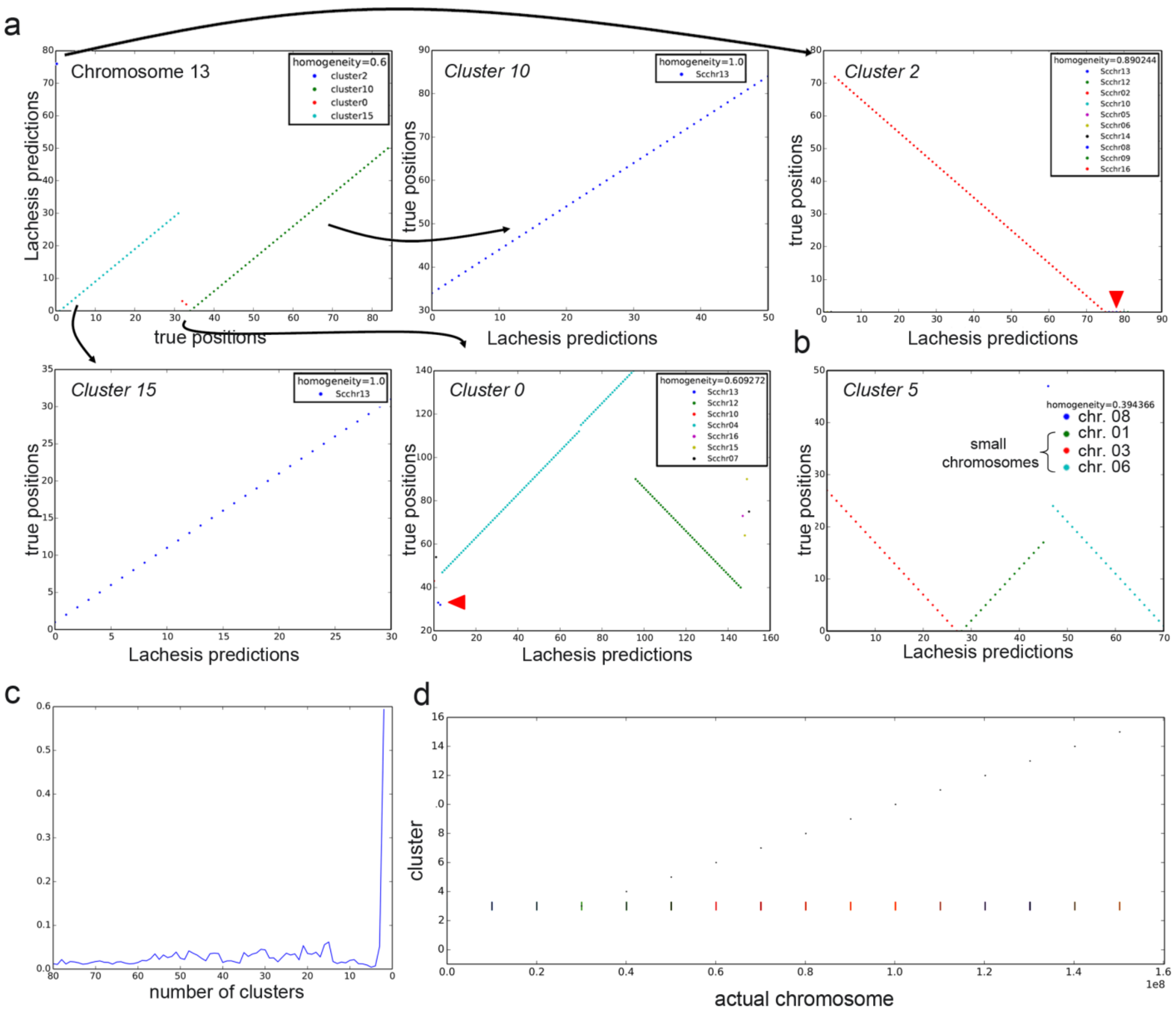


**Supplementary Figure 1**: Assembly of virtual *S. cerevisiae* contigs using Lachesis[1] and dnaTri[2]. (**a**) Example of inaccurate clustering by Lachesis, starting with the set of bins assembled by GRAAL in **Fig. 2**. Two large chromosomal segments of chromosome 13 were attributed to clusters 10 and 15, whereas two small regions of the same chromosome were incorporated in clusters 0 and 2 (red arrowheads). (**b**) Example of inaccurate clustering by Lachesis of small chromosomes 1, 3, and 6 into a single cluster. Note that although the 95% of the bins are correctly aligned with respect to their neighbors, this measure does not reflect the overall quality of the assembly. (**c**) dnaTri also fails to retrieve the correct number of yeast chromosomes when applied to yeast contact data. Both plots were generated by dnaTri. The left plot shows the average clustering step length as function of the number of clusters tested (see Figure 3a of the dnaTri paper[2]). The number of clusters chosen by dnaTri corresponds to the maximum of this

graph and in this case equals 2 instead of the expected number of 16 chromosomes. The right plot shows the assignment of contigs from the 16 chromosomes to clusters, revealing that the vast majority was incorrectly grouped into a single cluster.

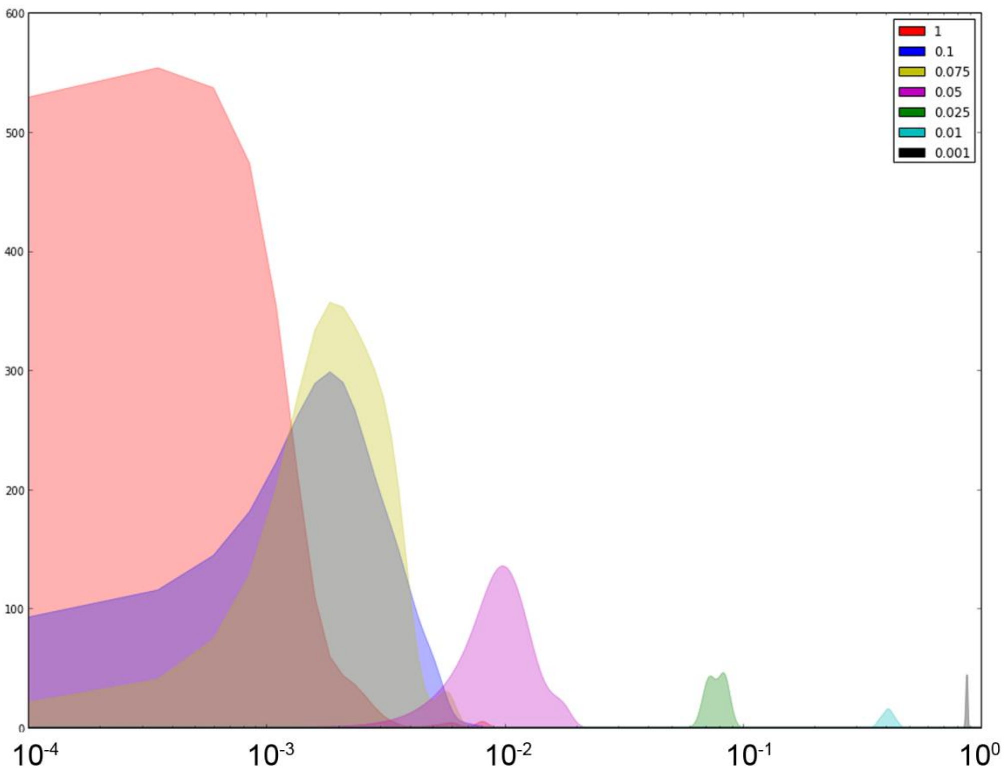


**Supplementary Figure 2:** Distribution of the error rate for sets of randomly down-sampled 3C dataset (from 1X to 0.001X for the *S. cerevisiae* matrix containing 21,457,486 contacts). The x-axis represents the error rate in log scale. For each down-sampled dataset, 15,000 iterations were performed.

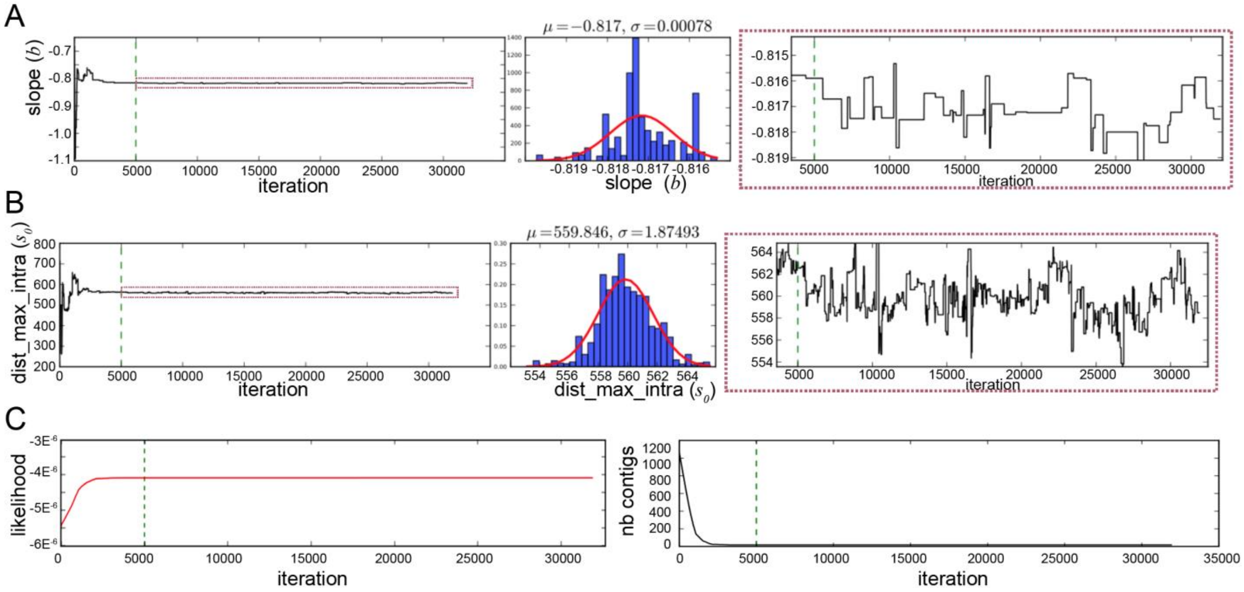


**Supplementary Figure 3: Evolution of the parameters** of the model (**a**) The slope reflects the intrachromosomal contact frequencies as a function of genomic separation (i.e. nuisance parameter *b* in the model; Material and Methods), repeatedly revisited over the 50,000 iterations in light of contact data. (**b**) Dist_max_intra represent the threshold, in kb, allowing discrimination between intra- and inter-chromosomal contacts, with inter-chromosomal frequencies assumed constant, corresponding to nuisance parameter $s_0$. Both the slope and the Dist_max_intra values are repeatedly reassessed based on the 3D data, fluctuating around an average value (μ), as illustrated by the close-ups (red (red dotted squares) on each curve. (**c**) Evolution of the likelihood and the number of contigs as function of the number of iterations.

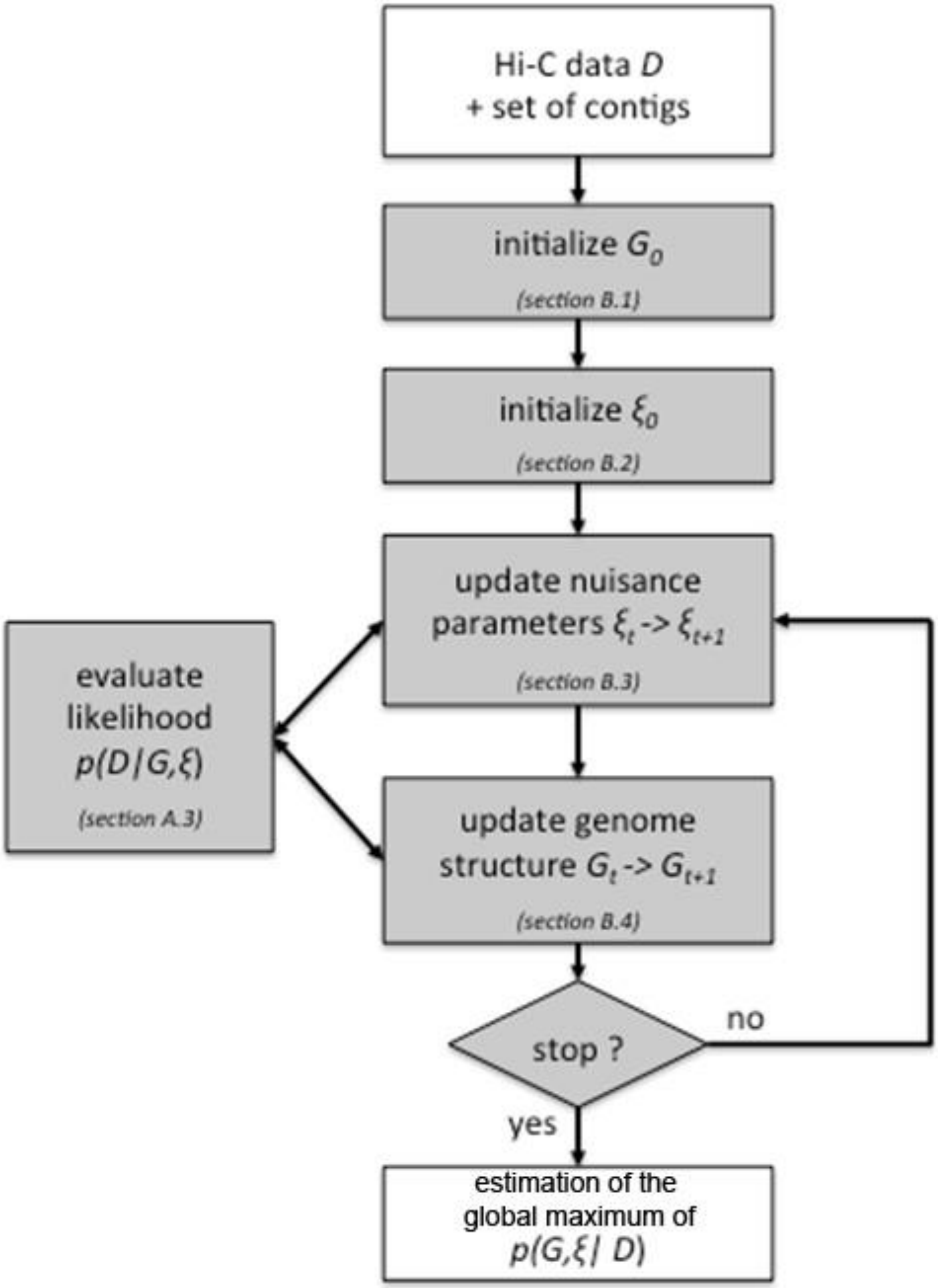


**Supplementary Figure 4: Overview of the GRAAL algorithm.**

**Supplementary Table 1. Features of three genome assembly algorithms based on 3D contact data**

| Feature | GRAAL | Lachesis[1] | dnaTri[2] |
|---|---|---|---|
| Predicts number of chromosomes | Yes | No | Yes |
| Corrects automatically initial misassemblies | Yes | No | No* |
| Orients contigs | Yes | Yes | No |
| Identifies repeated regions | Yes | No | No |
| Estimates assembly uniqueness | Yes | No | No** |

* not directly: the user can still cut the initializing contigs before the clustering step

** the probabilistic framework of the dnaTri algorithm is very elegant and allows it to estimate the likelihood of the structure, but, as acknowledged by the authors, there is no guarantee on the global optimum of the solution.

**Supplementary Table 2**: Sequencing adapters used in this study

| oligos | sequence | library |
|---|---|---|
| **MM70** | GTANNNNNNAGATCGGAAGAGCGGTTCAGCAGGAATGCCGAG | Malaysian yeast strain |
| **MM71** | ACACTCTTTCCCTACACGACGCTCTTCCGATCTNNNNNNTACT | |
| **MM182** | TCTNNNNNNAGATCGGAAGAGCGGTTCAGCAGGAATGCCGAG | YKF1246 yeast strain |
| **MM183** | ACACTCTTTCCCTACACGACGCTCTTCCGATCTNNNNNNAGAT | |
| **MM106** | TGGNNNNNNAGATCGGAAGAGCGGTTCAGCAGGAATGCCGAG | QM6a *T. reesei* strain |
| **MM107** | ACACTCTTTCCCTACACGACGCTCTTCCGATCTNNNNNNCCAT | |
| **MM108** | CCANNNNNNAGATCGGAAGAGCGGTTCAGCAGGAATGCCGAG | *E. histolytica* strain |
| **MM109** | ACACTCTTTCCCTACACGACGCTCTTCCGATCTNNNNNNTGGT | |

**Supplementary Table 3. Summary of the initialization parameters for the different analysis**

| Dataset | enzyme | nb of bins | n contacts | Mean nb of RFs per bin | mean bin size (kb) |
|---|---|---|---|---|---|
| *S. cerevisiae* | *Dnp*II | 1086 | 21457086 | 27 | 11 |
| *Trichoderma reesei* QM6a | *Dnp*II | 1193 | 15014468 | 27 | 27,9 |
| YKF1246 *S.c.* strain | *Dnp*II | 1295 | 21830579 | 27 | 9,5 |
| Malaysian *S.c.* strain | *Dnp*II | 3136 | 8353283 | 9 | 3,8 |
| Human chr7/17/19/22 | *Hind*III | 3607 | 19672219 | 9 | 95,9 |
| Human *de novo* chr14 | *Hind*III | 8382 | 1156115 | 3 | 8,8 |

## Supplementary Method

The following provides a more detailed description of the algorithm implemented in GRAAL.

The description of GRAAL can be divided into two main components: (i) the probabilistic model that assigns a likelihood to a given linear (one-dimensional) genome structure given a specific contact/Hi-C data set, and (ii) the sampling algorithm used to explore the space of linear genome structures (and nuisance parameters).

### A. Probabilistic model

A.1. Bayesian inference approach:

We consider the genome assembly problem as a Bayesian inference problem, taking inspiration from previous work in protein structure determination[3]. In its simplest form, the Bayes rule reads:

$$p(G|D) \propto p(D|G)p(G)$$

where $G$ denotes the linear genome structure to be determined, $D$ is the Hi-C data set (both will be defined more precisely below), $p(A|B)$ is the probability density of $A$ conditioned on $B$, and $\propto$ indicates proportionality. Our goal is to determine, or at least approximate, the posterior probability $p(G|D)$. The above formula provides a means to compute this probability density (up to a normalizing factor) given a probabilistic data generation model, $p(D|G)$ (called likelihood) and data-independent assumptions about the structure, encapsulated by the prior probability $p(G)$.

In practice, our data generation model involves several parameters (called nuisance parameters) that are not known *a priori* (see below). Therefore, we include these parameters, collectively noted as $\xi$, in the Bayesian formulation, yielding:

$$p(G,\xi|D) \propto p(D|G,\xi)p(G,\xi) = p(D|G,\xi)p(G)p(\xi)$$

where for the latter identity we assumed statistical independence of the genome structures $G$ and the nuisance parameters $\xi$.

We next assume that in absence of data, all possible genome structures and nuisance parameters are equally probable, i.e. that $p(G)$ and $p(\xi)$ are constants (flat priors). With these assumptions the Bayes rule reduces to:

$$p(G, \xi | D) \propto p(D | G, \xi)$$

To compute the likelihood $p(D|G, \xi)$ we need a data generation model that relates the contact frequencies measured by the Hi-C experiment to an assumed linear genome structure and the nuisance parameters.

A.2. Notations and definitions for the genome structure *G* and the Hi-C data *D*:

Before describing our model for $p(D|G, \xi)$ , we need a more formal definition of the variables *G*, and *D*. The parameters $\xi$ will be defined in section A.1.3.

Genome structure:

First, we define *G* as an unordered set of *N* contigs $\mathcal{C}_i$:

$$G = \{\mathcal{C}_1, \mathcal{C}_2, \cdots, \mathcal{C}_{N_c}\}$$

If the genome is perfectly assembled, each contig corresponds exactly to a single chromosome. Hi-C reads are mapped to restriction fragments $\mu_i$ defined by the restriction enzyme cutting sites. We therefore consider the restriction fragment $\mu_i$ as the elementary units of a genome assembly. However, many operations performed by GRAAL are not applied to individual restriction fragments, but to ordered sets of *p* consecutive fragments, which we call 'bins' and note :

$$f_k = (\mu_{k,1}, \ldots, \mu_{k,p})$$

Whenever possible, we choose $p = p_m$ where $p_m$ is a single user-defined constant typically set to 3. However, if the number of restriction fragments in a contig is not a multiple of $p_m$ , then some bins will consist of $p < p_m$ fragments.

We define a contig as an ordered sequence of bins, noted:

$$\mathcal{C}_k = \left(f_{\varphi_k^1}, f_{\varphi_k^2}, \ldots, f_{\varphi_k^{L_k}}\right)$$

where $\mathfrak{F}_G = \{f_1, f_2, \cdots, f_n\}$ is the set of all bins, $L_k$ is the number of bins in contig $\mathcal{C}_k$ and $\varphi_k$ is an indexing function with $\varphi_k^i \in [1, \ldots, n]$. The subscript in $\mathfrak{F}_G$ is used to indicate that the bins

rely on an initial assumed set of contigs $G_0$. We also introduce the two functions $\psi_1(i)$ and $\psi_2(i)$ such that:

$$\begin{cases} \psi_1(\varphi_k^i) = i \\ \psi_2(\varphi_k^i) = k \end{cases}$$

Next, we define $s_G(f_i, f_j)$ as the genomic distance (in units of base pairs) between two bins. This distance is obviously only defined for bins belonging to the same contig, i.e. for $\psi_2(i) = \psi_2(j)$. For $\psi_2(i) \neq \psi_2(j)$ we consider that $s_G(f_i, f_j) = \infty$.

<u>Hi-C/3C-seq contact data:</u>

The chromosome contact data used by GRAAL are obtained after mapping the Hi-C/3C-seq reads to an initial set of fragments $\mu_i, i = 1..N_f$. We define *D* as the matrix whose entries $D_{i,j}$, $\left((i,j) \in [1, \ldots, N_f]^2\right)$ are the number of Hi-C3C-seq reads pair mapped to each pair of fragments $(\mu_i, \mu_j)$. Please note that, although the sampling algorithm of GRAAL (described below) manipulates the genome structure at the level of super-contigs, the likelihood will always be evaluated by considering the contact data at the resolution of individual fragments.

<u>A.3. Likelihood and nuisance parameters:</u>

We now need a means to relate the probability of the matrix *D* to the assumed linear genome structure *G*. Our first step is to relate the probability of each entry $D_{i,j}$ to the contact probability between fragments $\mu_i$ and $\mu_j$, which we note $q_{i,j}$. Since $D_{i,j}$ results from a counting process, its probability can be modeled as a Poisson distribution:

$$\begin{cases} P(D_{i,j} = n) = \text{Poisson}(n; \lambda_{i,j}) \overset{\text{def}}{=} \begin{cases} \dfrac{\lambda_{i,j}^n}{n!} e^{-\lambda_{i,j}} \text{ for } n \in \mathbb{N} \\ 0 \text{ otherwise} \end{cases} \\ \lambda_{i,j} = N_D \, q_{i,j} \end{cases}$$

where $N_D$ is the total number of independent counts. Although $N_D$ is strictly speaking also a random number (which depends chiefly on sequencing depth and criteria used to validate the read pairs), for simplicity we treat it as a constant and simply set: $N_D = \sum_{i=1}^{N_f} \sum_{j=i}^{N_f} D_{i,j}$. We further assume that contacts between distinct pairs of fragments are independent from each other, such that :

$$p(D) = \prod_{i=1}^{n} \prod_{j=i}^{n} p(D_{i,j})$$

which implies:

$$p(D) = \prod_{i=1}^{n} \prod_{j=i}^{n} \text{Poisson}(D_{i,j}; N_D\, q_{i,j})$$

In order to be able to calculate $p(D|G, \xi)$, we now need to relate the contact probabilities $q_{i,j}$ to $G$ and $\xi$.

Contact probabilities are intimately dependent on how chromosomes are folded and positioned relative to each other. The detailed relationship between the 3D and 1D architecture of the genome is in general complex, depends on the organism and cell state, and is subject of much current research (e.g. [4]). Nevertheless, some important features are present in all Hi-C/3C-seq data sets obtained so far, and are also in good agreement with predictions from polymer physics. Specifically, the contact probability $P_{cis}$ between loci on the same chromosome (*cis* contacts) decays as a power law with increasing genomic distances $s$ (as expressed in bp). This relation holds up to a genomic distance $s_0$ above which contact probabilities are approximately constant, i.e.:

$$\begin{cases} P_{cis}(s) = P_t \left(\dfrac{s}{s_0}\right)^b & \text{for} \quad s \le s_0 \\ P_{cis}(s) = P_t & \text{for} \quad s \ge s_0 \end{cases}$$

These relationships have been approximately verified in a number of different organisms, with variable values for $b$ , $s_0$, and $P_t$, and can be recapitulated by computational simulations of polymer dynamics [5–9].

The contact probabilities between loci on distinct chromosomes (*trans* contacts) are less amenable to simple theoretical predictions and arguably more sensitive to biological specificities such as organism and cell type. They are also on average much weaker than *cis* contact probabilities. For simplicity and generality, we therefore simply assume that *trans* contacts have the same probability as long-range *cis* contacts:

$$P_{trans} = P_t$$

Our probabilistic model is therefore characterized by only 3 parameters, collectively noted as $\xi$:

$$\xi = (P_t, s_0, b).$$

Because these parameters cannot reliably be predicted *a priori*, they will be sampled together with $G$ as will be detailed below.

With the equations above, we now have all ingredients to calculate $p(D|G,\xi)$:

$$p(D|G,\xi) = \prod_{i=1}^{n} \prod_{j=i}^{n} \mathrm{Poisson}\left(D_{i,j}; N_D\, q_{i,j}\right) \qquad \text{(Eq 1)}$$

with:

$$\boldsymbol{p(q_{i,j}|G,\xi) = g(s_G(i,j); P_t, s_0, b)} \overset{\text{def}}{=} \begin{cases} \boldsymbol{P_t \left(\frac{s_G(i,j)}{s_0}\right)^{b}} & \text{if} \quad \boldsymbol{s_G(i,j) \le s_0} \\ \boldsymbol{P_t} & \text{otherwise} \end{cases} \qquad \text{(Eq 2)}$$

**B. Sampling algorithm**

The above formulas allow us to compute the posterior probability of any assumed linear genome structure $G$ (and the nuisance parameters $\xi$) given $D$, a contact data set obtained by mapping 3C-seq/Hi-C reads to an initial set of contigs. In order to explore the entire probability density $p(G,\xi)$, we need a method to sample the extremely large (or infinite) space of possible linear genome structures. For this purpose, we implemented an algorithm inspired by the Markov-Chain Monte-Carlo (MCMC) Gibbs sampler. Starting from an initialization $(G_0, \xi_0)$ the algorithm makes a large number of random moves across the space $(G\ \ ,\xi\ \ )$ to be sampled, and uses a probabilistic rule to accept or reject individual moves $(G_t,\xi_t) \to (G_{t+1},\xi_{t+1})$. After a sufficient number of steps, once the chain has reached equilibrium, a subset of the accepted samples can be used to approximate the global maximum of the probability density $p(G,\xi)$. An overview of the algorithm's main modules is provided in .

At each iteration, GRAAL updates first nuisance parameters $\xi_t$ and then the genome structure $G_t$. Below, we separately describe first the initialization of $G_{t=0}$ and $\xi_{t=0}$ and then the update rules for $G_t$ and $\xi_t$.

B.1. Initialization of the genome, $G_0$

Different initializations can be considered for the initial set of contigs $G_0$ depending on the availability of a preliminary assembly of the organism under study, or of a related genome. The initial set of contigs does not need to be perfect, since GRAAL can split and rearrange incorrectly assembled contigs. However, in our current implementation, the restriction fragments $\mu_i$ and the bins $f_i$ , whose definition depends on $G_0$, cannot be broken. In our paper, we considered the following different types of initializations:

- The reference budding yeast genome (16 chromosomes; GCF_000146045.1) was used as validation data, since a high quality assembly of this genome is already available. In order to simulate an incomplete assembly of this genome, we split the genome into $N_c$ =1,086 bins (of approximately 11Kb) to initialize GRAAL.
- For YFK1246, the structural mutant of budding yeast[10], $N_c$ =3,171 bins of 9 RFs (*Dpn*II restriction enzyme) of the reference budding yeast genome were used to initialize GRAAL.
- We also used this initialization to assemble the Malaysian budding yeast isolate (UWOPS03-461.4).
- The *Trichoderma* genome (*ATCC* 13631)[11] of strain QM6a was only partly assembled (including using long-insert paired-end data), yielding 77 scaffolds. Rather than initializing $G_0$ with these scaffolds, those were split into bins of 81 RF, which led to $N_c = 1193$ bins that were used to initialize GRAAL.
- For the human chromosome 14, we downloaded the 4,722 contigs obtained from the ALLPATHS-LG *de novo* assembly (average siz e 20kb)12. A filter was applied to identify RFs (from the HindIII restriction enzyme used in the Hi-C experiment13) presenting little or no read coverage. If reads appears sparse along a RF compared to the distribution of read coverage over the entire population of RFs, the RF was discarded. If the entire contig appeared undercovered, it was therefore discarded. A similar filtering step is used by dnaTri2. We then split the remaining 2,917 contigs into bins of 3 RFs.

As a general strategy to complete the assembly of an imperfectly assembled genome, we recommend starting from the existing contigs and splitting them into bins as illustrated here for *Trichoderma.* The user of GRAAL has the option to choose whether to split these contigs or not (see section B).

B.2. Initialization of the nuisance parameters, $\xi_0$

The initial values of the parameters $\xi_0 = (P_t, s_0, b)$ are obtained based on the Hi-C data $D$ and the initial genome structure $G_0$ as follows:

First, the initial value of $P_t$ is set to the contact probability averaged over all pairs of bins belonging to different contigs, i.e.:

$$P_t = \frac{\sum_{i<j}\left(1-\delta_{\psi_2(i),\psi_2(j)}\right)D_{i,j}}{\sum_{i<j}\left(1-\delta_{\psi_2(i),\psi_2(j)}\right)}$$

where $\delta_{i,j} = 1$ if $i$=$j$ and $\delta_{i,j} = 0$ otherwise.

Next, we construct a histogram of *cis*-contact frequencies with genomic intervals $[d_0, \dots d_M]$, ranging from $d_0$ =0 to $d_M = \max\,(L_k)_{k=1..N_c}$, the length of the longest contig in $G_0$. For each genomic bin $[d_l, d_{l+1}]$, the histogram reports the mean contact frequency, among all $N_c$ contigs, between bins sharing a contig and separated by genomic distances $s \in [d_l, d_{l+1}]$:

$$F_l = \frac{\sum_{i<j}\delta_{\psi_2(i),\psi_2(j)}H\left(s_{G_0}(f_i,f_j)-d_l\right)H\left(d_{l+1}-s_{G_0}(f_i,f_j)\right)D_{i,j}}{\sum_{i<j}\delta_{\psi_2(i),\psi_2(j)}H\left(s_{G_0}(f_i,f_j)-d_{li}\right)H\left(d_{l+1}-s_{G_0}(f_i,f_j)\right)}$$

where H is the Heaviside function ($H(x) = 1$ for $x \geq 0$ and $H(x) = 0$ otherwise) . We then estimate the initial values of $s_0$ and $b$ by least squares fitting of (Eq1) to $F_i$, i.e.:

$$(s_0, b)=\arg\min \sum_{l=1}^{M}\left(F_l - g\left(\tfrac{1}{2}(d_l+d_{l+1}); P_t, s_0, b\right)\right)^2$$

This minimization is performed using a quasi-Newton method[12].

B.3. Monte Carlo modifications of the genome $G_t \rightarrow G_{t+1}$:

B.3.1. Virtual mutations:

We will call 'virtual mutations' the random changes applied to the genome structure. GRAAL considers 5 different types of elementary virtual mutations and 4 composite mutations as detailed below.

Elementary mutations:

The 5 elementary mutations are defined as follows:

- Split: this mutation splits a contig at a bin and is formally noted as $\mathcal{S}(i,\varepsilon)$, where $i$ is the index of the bin $f_i$, and $\varepsilon = \pm 1$ indicates whether the split occurs to the left or right of the bin. As a result of this operation, contig $\mathcal{C}_{\psi_2(i)}$ is replaced by two new contigs:

$$\begin{cases} \begin{cases} \mathcal{C}_{\text{new,left}} = \left(f_{\varphi_k^1}, \ldots, f_{\varphi_k^{l-1}}\right) & \text{if } \varepsilon = -1 \\ \mathcal{C}_{\text{new,left}} = \left(f_{\varphi_k^1}, \ldots, f_{\varphi_k^{l}}\right) & \text{if } \varepsilon = +1 \end{cases} \\ \begin{cases} \mathcal{C}_{\text{new,right}} = \left(f_{\varphi_k^l}, \ldots f_{\varphi_k^{L_k}}\right) & \text{if } \varepsilon = -1 \\ \mathcal{C}_{\text{new,right}} = \left(f_{\varphi_k^{l+1}}, \ldots f_{\varphi_k^{L_k}}\right) & \text{if } \varepsilon = +1 \end{cases} \\ G_{\text{new}} = G \setminus \mathcal{C}_{\psi_2(i)} \cup \mathcal{C}_{\text{new,left}} \cup \mathcal{C}_{\text{new,right}} \end{cases}$$

where $(l,k) = (\psi_1(i), \psi_2(i))$.

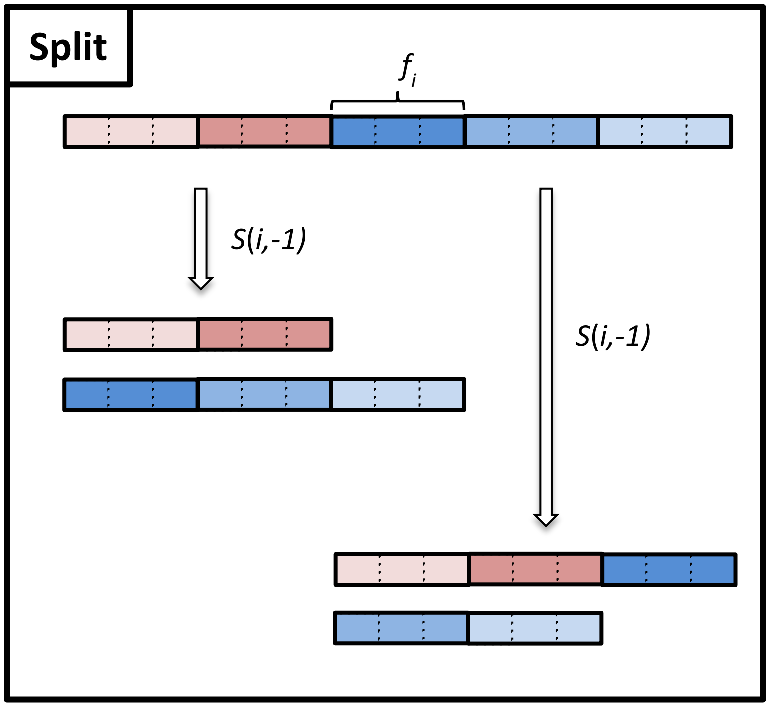


- Paste: this mutation concatenates two contigs and is formally noted as $\mathcal{P}(k,l,\varepsilon)$, where $k$ and $l$ are the indices of the two contigs to be pasted, and $\varepsilon = \pm 1$ indicates whether or not contig $\mathcal{C}_l$ is flipped before pasting. As a result of this mutation, the two contigs $\mathcal{C}_k$ and $\mathcal{C}_l$ are replaced by a single new contig obtained by concatenating $\mathcal{C}_l$ (or it flipped version) to the right of $\mathcal{C}_k$. Note that the orientation of the bins inside each contig are preserved.

$$\begin{cases} \mathcal{C}_{\text{new}} = \left(f_{\varphi_k^1}, f_{\varphi_k^2}, \ldots, f_{\varphi_k^{L_k}}, f_{\varphi_l^1}, f_{\varphi_l^2}, \ldots, f_{\varphi_l^{L_l}}\right) & \text{if } \varepsilon = +1 \\ \mathcal{C}_{\text{new}} = \left(f_{\varphi_k^1}, f_{\varphi_k^2}, \ldots, f_{\varphi_k^{L_k}}, \mathcal{F}\left(f_{\varphi_l^{L_l}}\right), \mathcal{F}\left(f_{\varphi_l^{L_l-1}}\right), \ldots, \mathcal{F}\left(f_{\varphi_l^1}\right)\right) & \text{if } \varepsilon = -1 \\ G_{\text{new}} = G \setminus \{\mathcal{C}_k, \mathcal{C}_l\} \cup \mathcal{C}_{\text{new}} & \end{cases}$$

where the operator $\mathcal{F}$ (flipping a bin) is defined below.

Split and paste are reciprocal operations, i.e. one mutation can reverse the effect of the other, such that: $\mathcal{P}(k_i^-, k_i^+, -1) \circ \mathcal{S}(i, \varepsilon_1) = \mathcal{S}(i_l, \varepsilon_1) \circ \mathcal{P}(k, l, +1) = \mathcal{N}$, where $k_i^-$ is the index of the contig resulting from the split operation that was originally to the left of bin $f_i$ , $k_i^+ = \psi_2(i)$ is the index of the contig still containing $f_i$ after the split, $i_{k,l}$ is the leftmost bin of contig $l$, and $\mathcal{N}$ is the "null" mutation, which leaves the genome unchanged.

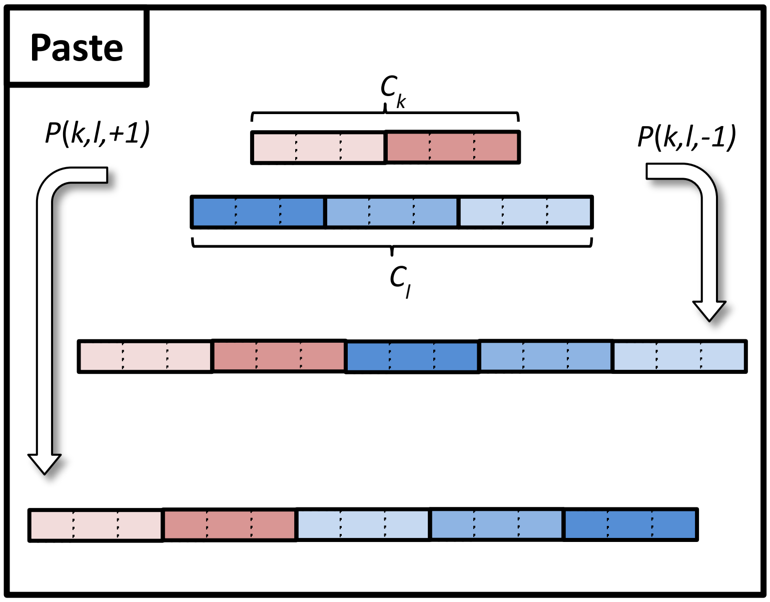


- <u>Duplicate</u>: This mutation duplicates a bin $f_i$ and is formally noted as $\mathcal{D}(i)$. As a result of this mutation, a copy of $f_i$ is added to the current set of bins and a new contig consisting of this single bin is added to the current contig set :

$$\begin{cases} \mathfrak{F}_{\text{new}} = \mathfrak{F} \cup \{f_i\} \\ G_{\text{new}} = G \cup \{f_i\} \end{cases}$$

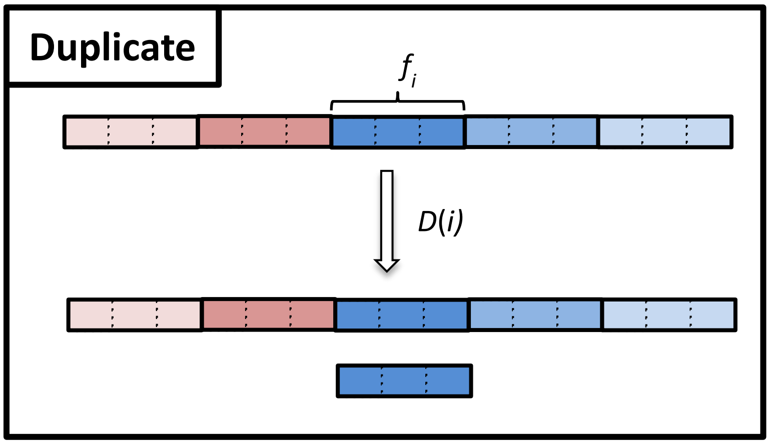


- Delete: formally noted as $\mathcal{R}(i)$, this mutation leads to the removal of bin $f_i$ from the current set of bins $\mathfrak{F}_t$ and from the contig that contained it:

$$\begin{cases} \mathfrak{F}_{\text{new}} = \mathfrak{F} \quad \setminus \{f_i\} \\ \mathcal{C}_{k,\text{new}} = \left(f_{\varphi_k^1}, \dots, f_{\varphi_k^{l-1}}, f_{\varphi_k^{l+1}}, \dots f_{\varphi_k^{L_k}}\right) \\ G_{\text{new}} = G \quad \setminus \{\mathcal{C}_k\} \cup \mathcal{C}_{k,\text{new}} \end{cases}$$

where $(l, k) = (\psi_1(i), \psi_2(i))$.

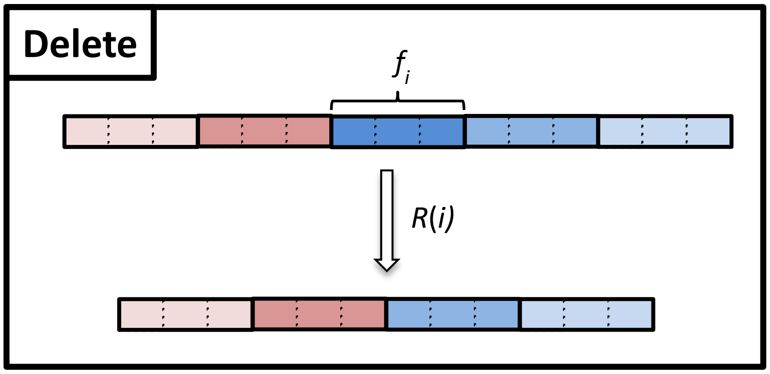


- Flip: formally noted as $\mathcal{F}(i)$, this mutation flips the orientation of bin $f_i$ in its containing contig.

$$\begin{cases} f_{\varphi_k^l,\text{new}} = (\mu_{k,p}, \mu_{k,p-1}, \dots, \mu_{k,1}) \\ \mathfrak{F}_{\text{new}} = \mathfrak{F} \quad \setminus \left\{f_{\varphi_k^l}\right\} \cup \left\{f_{\varphi_k^l}\right\} \\ \mathcal{C}_{k,\text{new}} = \left(f_{\varphi_k^1}, \dots, f_{\varphi_k^{l-1}}, f_{\varphi_k^l,\text{new}}, f_{\varphi_k^{l+1}}, \dots f_{\varphi_k^{L_k}}\right) \\ G_{\text{new}} = G \quad \setminus \{\mathcal{C}_k\} \cup \mathcal{C}_{k,\text{new}} \end{cases}$$

where $(l, k) = (\psi_1(i), \psi_2(i))$. The reciprocal operation of a flip is itself: $\mathcal{F}(i) \circ \mathcal{F}(i) = \mathcal{N}$.

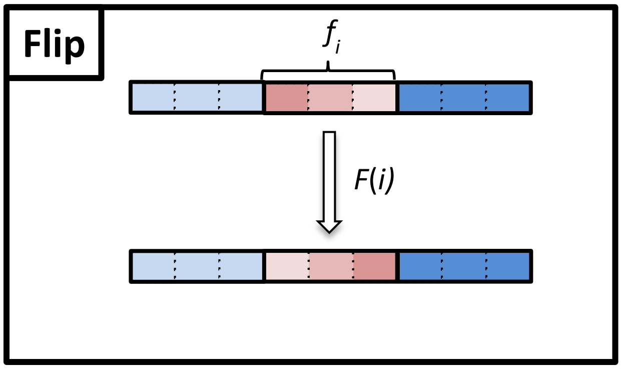


Any complex alteration of the genome (defined at the resolution of bins) can be decomposed into a sequence of these five mutations $\mathcal{S}, \mathcal{P}, \mathcal{D}, \mathcal{R},$ and $\mathcal{F}$. However, for complex structural changes such as translocations, the required sequence may be very long, and it might take unreasonable time for the sampler to achieve them using Monte Carlo moves. Therefore, we introduce the following composite mutations:

<u>Composite mutations:</u>

- <u>Eject</u>: this mutation, noted as $\mathcal{E}(i)$, pops out bin $f_i$ from its contig, and pastes together the two extremities flanking the bin, leaving $f_i$ as a new contig. It is therefore a composite of two split and one paste mutations:

$$\mathcal{E}(i) = \mathcal{P}(k_i^-, k_i^+, +1) \circ \mathcal{S}(i, +1) \circ \mathcal{S}(i, -1)$$

  where $k_i^-$ and $k_i^+$ are the indices of the contigs resulting from the two splits and originally located to the left and the right of bin $f_i$.

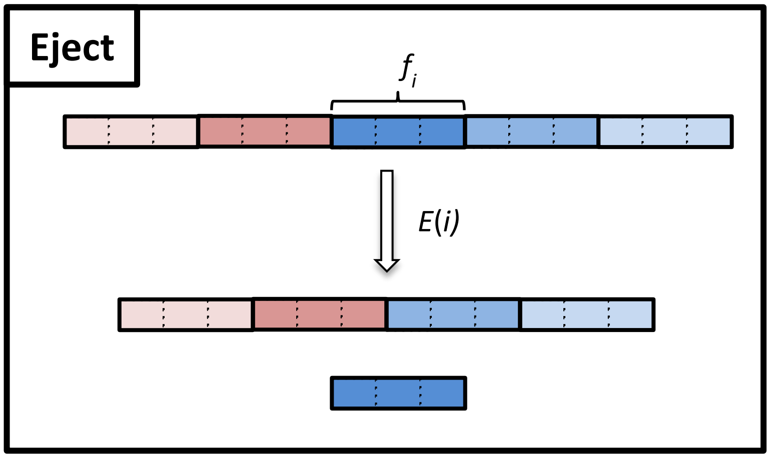

- Insert: this mutation, noted as $\mathcal{I}(i,j)$ inserts an isolated bin $f_i$ (i.e. a contig consisting of a single bin) to the right of bin $f_j$ into its contig $\mathcal{C}_{\psi_2(j)}$. It is a composite of one split and two paste mutations:

$$\mathcal{I}(i,j) = \mathcal{P}(i,k_j) \circ \mathcal{P}(j,i,+1) \circ \mathcal{S}(j,+1)$$

  where $k_j = \psi_1(j) + 1$ designates the bin immediately to the right of $f_j$ within contig $\mathcal{C}_{\psi_2(j)}$. Ejection and insertions are reciprocal operations, i.e. : $\mathcal{I}(i,j) \circ \mathcal{E}(i) = \mathcal{E}(i) \circ \mathcal{I}(i,j) = \mathcal{N}$.

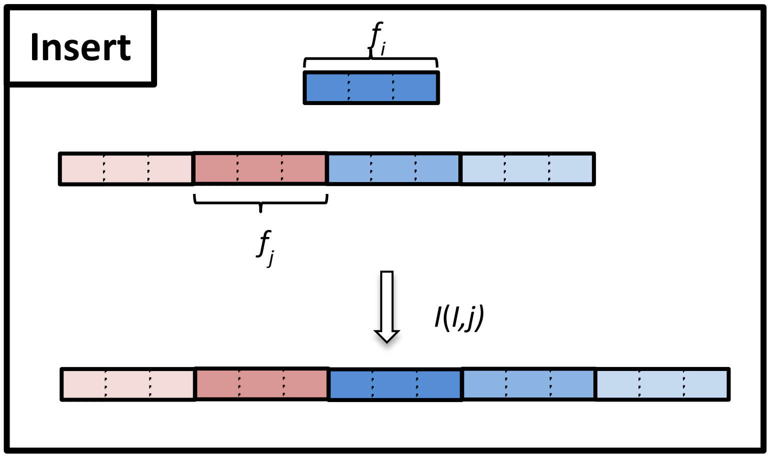


- Translocate: this mutation mimics a biological translocation which swaps two parts of distinct chromosomes and is denoted as $\mathcal{T}(i,j,\varepsilon_1,\varepsilon_2)$, where $i$ and $j$ designate the bin on the two contigs $\mathcal{C}_{\psi_2(i)}$ and $\mathcal{C}_{\psi_2(j)}$, to the right of which the translocation events take place, and where $\varepsilon_1 = \pm 1$ and $\varepsilon_2 = \pm 1$ indicate whether the two swapped regions are flipped or not. This operation is a composite of two split and two paste mutations:

$$\mathcal{T}(i,j,\varepsilon_1,\varepsilon_2) = \mathcal{P}(j,k_j^+,\varepsilon_2) \circ \mathcal{P}(i,k_i^+,\varepsilon_1) \circ \mathcal{S}(j,+1) \circ \mathcal{S}(i,+1)$$

  where $k_i = \psi_1(i) + 1$ and $k_j = \psi_1(j) + 1$ are the indices of the bin immediately to the right of $f_i$ and $f_j$, respectively, on contigs $\mathcal{C}_{\psi_2(i)}$ and $\mathcal{C}_{\psi_2(j)}$.

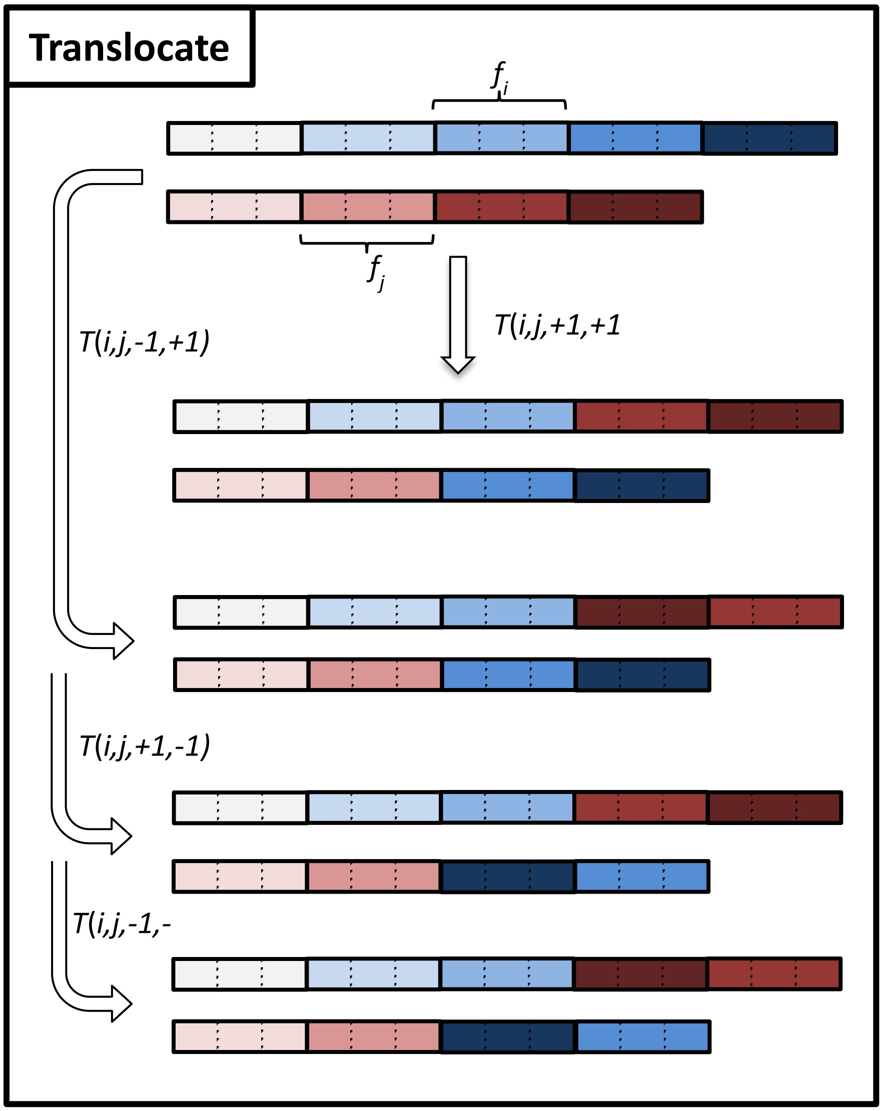


The reciprocal operation of a translocation is itself:

$$\mathcal{T}(i,j,\varepsilon_1,\varepsilon_2) \circ \mathcal{T}(i,j,\varepsilon_1,\varepsilon_2) = \mathcal{N}$$

- Jump: this mutation, noted $\mathcal{J}(i,j)$, extracts bin $f_i$ from its contig $\mathcal{C}_{\psi_2(i)}$ and inserts it to the right of bin $f_j$ on contig $\mathcal{C}_{\psi_2(j)}$. It can be decomposed into an ejection followed by an insertion:

$$\mathcal{J}(i,j) = \mathcal{I}(i,j) \circ \mathcal{E}(i)$$

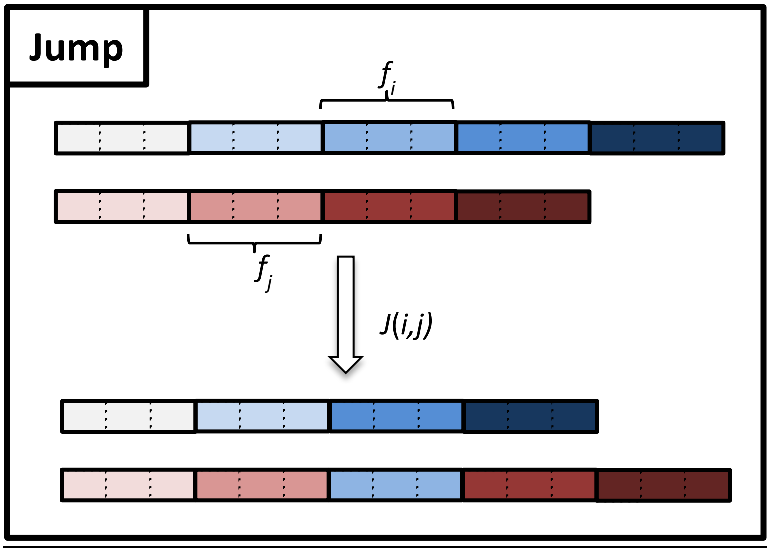


These composite mutations can generate more complex and drastic alterations of genome structure in a single step, thereby allowing faster exploration of larger regions of structure space than the elementary mutations.

We now introduce some notations that will be important for the following section. First, we call $\mathcal{M} = \{\mathcal{P}, \mathcal{S}, \mathcal{D}, \mathcal{R}, \mathcal{F}, \mathcal{E}, \mathcal{I}, \mathcal{T}, \mathcal{J}\}$ the set of all 9 mutations and use the generic notation $\Theta_i \in \mathcal{M}$ with $i \in \{1,2,..,9\}$ for individual members of this set (for example, $\Theta_2$ is the paste mutation. We point out that each of the 9 mutations can be defined by either one (mutations $\mathcal{S}, \mathcal{D}, \mathcal{R}, \mathcal{F}, \mathcal{E}$) or two indices of bins (mutations $\mathcal{P}, \mathcal{I}, \mathcal{T}, \mathcal{J}$) and, for some mutations, one or two auxiliary binary parameters $\varepsilon_i = \pm 1$. To formally note the parameters of an arbitrary mutation $\Theta_k$ in $\mathcal{M}$, we can therefore use the notation: $\Theta_k(i, j, \alpha)$, where it is understood that $j$ is relevant only for mutations $\mathcal{P}, \mathcal{I}, \mathcal{T}, \mathcal{J}$ and $\alpha$ corresponds to the auxiliary parameter, if relevant (e.g. $\alpha = (\varepsilon_1, \varepsilon_2)$ for $\Theta_8 = \mathcal{T}$, $\alpha = \{\emptyset\}$ for $\Theta_3 = \mathcal{D}$). We call $\mathcal{A}_k$ the set of all possible values of the auxiliary parameter for mutation $\Theta_k$. For example, $\mathcal{A}_8 = \{(-1,-1), (-1,+1), (+1,-1), (+1,+1)\}$. Finally, we note $G^* = \Theta_k(G)$ the structure resulting from application of mutation $\Theta_k$ to the genome $G$.

B.3.2. Multiple Try Metropolis updates of genome structure

Now that we have defined the possible mutations, we explain how they are used to update genome structures.

In devising the sampling algorithm, we initially implemented a basic Metropolis-Hastings algorithm[12]. However, this led to very low acceptance rates of individual moves and excessive computation time. In order to accelerate the sampling, we therefore implemented a new algorithm based on a more sophisticated sampling strategy known as Multiple-Try Metropolis that evaluates several candidate moves at each step and has been shown to allow significantly improved computation times[13].

The canonical MTM method works as follow:

1. Randomly pick one bin $f_i$ by choosing a random integer *i* between 1 and N (the current number of bins) with uniform probability.
2. Next, randomly pick a number *K* of distinct bins $(f_j)_{j=1..K}$ with $f_j \neq f_i$. In contrast to the first bin $f_i$, however, these bins are not drawn with uniform probability, but with a probability:

$$V_i(j) = \frac{D_{i,j}}{\sum_{k\in[1,N] \quad ,k\neq l} D_{i,k}}$$

   As a consequence, the sampled bins $f_i$ tend to have high contact probability with $f_i$ and are therefore likely to be located in close linear proximity vicinity on the same chromosome.
3. Consider the set $\mathfrak{G}$ of all candidate genome structures $\Gamma_l$ obtained by separately applying each of the 9 mutations $\Theta_k$ to the current genome structure $G_t$ with all possible values of the auxiliary parameters, i.e.:

$$\mathfrak{G} = (\Gamma_l \ ) = \{\Theta_k(i,j,\alpha)(G_t \ );\ k \in [1,9], j = [1,K], \alpha \in \mathcal{A}_k\}$$

   Among all structures in this set, we pick a random subset of $K_1$ structures (with uniform probability):

$$\mathfrak{G}_{K_1} = \{\Gamma_1 \ , \ldots, \Gamma_{K_1}\}$$

   For each of these candidate structure, we evaluate the likelihood $\pi(\Gamma_l \ ) = p\left(D|\Gamma_l \ , \xi_t \ \right)$ using equations 1 and 2. Note that the nuisance parameters are held constant (they are updated separately as described in section A.2.4).
4. For each candidate structure $\Gamma_l \ = \Theta_k(i,j,\alpha)(G_t \ )$, we define:

$$w(G_t, \Gamma_l) = \pi(G_t) T(G_t, \Gamma_l)$$

where the proposal function $T$ is chosen as:

$$T(G_t, \Gamma_l) = V_i(j)$$

5. Among the $K_1$ proposed candidate structures, we select one, called $\Gamma$ with probability proportional to

$$w(\Gamma, G_t) = \pi(\Gamma) T(\Gamma, G_t) = \pi(\Gamma) V_j(i)$$

6. We note $j$ the index of the bin $f_j$ that led to this structure $\Gamma = \Theta_k(i, j, \alpha)(G_t)$. We then randomly pick another set of K bins $f_p$, with probability $V_j(p)$ and define a new set of genome structures :

$$\mathfrak{G}^* = (G_l^*) = \{\Theta_k(j, p, \alpha)(\Gamma);\ k \in [1,9], p = [1, K], \alpha \in \mathcal{A}_k\}$$

Among this set, we randomly pick (with uniform probability) $K_1 - 1$ structures.

$$\mathfrak{G}_{K_1-1}^* = \{G_1^*, \ldots, G_{K_1-1}^*\}$$

7. Finally, we compute the generalized Metropolis-Hastings acceptance ratio as:

$$r = \min\left\{1, \frac{w(\Gamma_1, G_t) + w(\Gamma_2, G_t) + \ldots + w(\Gamma_{K_1}, G_t)}{w(G_1^*, \Gamma) + \ldots + w(G_{K_1-1}^*, \Gamma) + w(G_{K_1}^*, \Gamma)}\right\}$$

With probability $r$, we accept the new structure $\Gamma$ and set $G_{t+1} = \Gamma$. In case of rejection, we set: $G_{t+1} = G_t$

However, in order to lower the computing load of the process we implemented an alternative version of the algorithm. At step 5, we set $G_{t+1} = \Gamma$ and therefore skip steps 6 and 7. The resulting random process is no longer a time homogeneous Markov chain, but the efficiency of this strategy is experimentally verified.

B.4. Monte Carlo updates of the nuisance parameters $\xi_t \to \xi_{t+1}$:

The nuisance parameters are updated as follows:

First, we randomly pick one of the three parameters with equal probability 1/3, i.e. we choose $\theta \in \{P_t, s_0, b\}$. Second, we consider a new candidate value for this parameter by addition of a normally distributed random variable:

$$\theta^* = \theta_t + \Delta\theta \quad \text{with} \quad \Delta\theta \sim \mathcal{N}(0, \sigma_\theta)$$

We chose to set the variance of the parameter change to a small fraction of the initial value: $\sigma_\theta = 10^{-4}\, \theta_{t=0}$. This choice was made because of the high sensitivity of the likelihood to small variations of the parameters.

We note $\xi^*$ the new candidate set of parameters obtained by replacing parameter $\theta$ by $\theta^*$ in $\xi_t$.

Next, we accept this candidate with probability:

$$r = \min\left(1, \frac{p(D|G_t, \xi^*)}{p\big(D|G_t, \xi\quad\big)}\right)$$

where the ratio of likelihoods on the right is computed using Eqs 1 and 2.

If this move is accepted, we set: $\xi_{t+1} = \xi^*$, otherwise we keep: $\xi_{t+1} = \xi_t$.

## B.5. Sampling from the Markov chain:

Starting from the initialization of $G_0$ and $\xi_0$ as defined in A.2.1 and A.2.2, we let the Markov moves update $G_t$ and $\xi_t$ under the rules specified in sections A.2.3 and A.2.4 for a total number of iterations $N_{\max}$. In order to approximate the probability distribution $p(G, \xi|D)$, we discard all samples obtained during an initial burn-in period specified by a number of iterations $N_{\text{burn-in}}$ and use all samples thereafter, i.e. we use $(G_t, \xi_t)$ with $N_{\text{burn-in}} \leq t \leq N_{\max}$. We chose $N_{\max}$ and $N_{\text{burn-in}}$ depending on $N_f$, the number of restriction fragments in the Hi-C data set $D$. Typically used values are: $N_{\text{burn-in}} = 3\, N_f$ and $N_{\max} = 10\, N_f$.

## B.6. Metrics

We use different metrics to quantify assembly quality or otherwise characterize the sampled structure probability density.

- $\text{iqr}\big(N_{\text{contigs}}\big)$ : One simple way to measure the variability among the sampled structures is to measure the variability of contig number. Here, we use the interquartile range (i.e. the difference between the 75% and the 25% percentiles) of the number of contigs in the structure samples $\left(G_{N_{\text{burn-in}}}, \ldots, G_{N_{\max}}\right)$.
- Error: In order to quantify the quality of assembly on a known genome, we define an error measured as follows: we examine the position of each bin $f_i$, $i = 1..N_f$ and ask if its immediate flanking neighbors and its orientation are correct. Depending on the answer,

we attribute a bin error $E_i \in \{0,1,2,3\}$ , where $E_i = 0$ if both neighbors and orientation are correct, and $E_i = 3$ if all are incorrect. We then define the total normalized error as $E = \frac{\sum_{i=1}^{N_f} E_i}{3N_f}$. The normalization ensures that $0 \leq E \leq 1$. A perfect assembly (at the level of bins) yields $E = 0$. Note that this measure is quite sensitive to assembly errors, since any displacement of a bin from its true position (irrespective of the magnitude of this displacement) and any incorrect orientation will increase $E$.

**Supplementary references**


1. Burton, J. N. *et al.* Chromosome-scale scaffolding of *de novo* genome assemblies based on chromatin interactions. *Nat. Biotechnol.* **31,** 1119–1125 (2013).
2. Kaplan, N. & Dekker, J. High-throughput genome scaffolding from in vivo DNA interaction frequency. *Nat. Biotechnol.* **31,** 1143–1147 (2013).
3. Rieping, W., Habeck, M. & Nilges, M. Inferential structure determination. *Science* **309,** 303 (2005).
4. Rosa, A. & Zimmer, C. Computational models of large-scale genome architecture. *Int. Rev. Cell Mol. Biol.* **307,** 275–349 (2014).
5. Barbieri, M. *et al.* Complexity of chromatin folding is captured by the strings and binders switch model. *Proc. Natl. Acad. Sci. U. S. A.* **109,** 16173–8 (2012).
6. Lieberman-Aiden, E. *et al.* Comprehensive mapping of long-range interactions reveals folding principles of the human genome. *Science* **326,** 289–93 (2009).
7. Duan, Z. *et al.* A three-dimensional model of the yeast genome. *Nature* **465,** 363–367 (2010).
8. Wong, H. *et al.* A predictive computational model of the dynamic 3D interphase yeast nucleus. *Curr. Biol. CB* **22,** 1881–90 (2012).
9. Tanizawa, H. *et al.* Mapping of long-range associations throughout the fission yeast genome reveals global genome organization linked to transcriptional regulation. *Nucleic Acids Res.* **38,** 8164–77 (2010).
10. Koszul, R., Caburet, S., Dujon, B. & Fischer, G. Eucaryotic genome evolution through the spontaneous duplication of large chromosomal segments. *EMBO J.* **23,** 234–243 (2004).
11. Martinez, D. *et al.* Genome sequencing and analysis of the biomass-degrading fungus *Trichoderma reesei* (syn. *Hypocrea jecorina*). *Nat. Biotechnol.* **26,** 553–560 (2008)
12. Salzberg, S. L. *et al.* GAGE: A critical evaluation of genome assemblies and assembly algorithms. *Genome Res.* **22**, 557–567 (2011).
13. Dixon, J. R. *et al.* Topological domains in mammalian genomes identified by analysis of chromatin interactions. *Nature* **485,** 376–380 (2012).
14. Press, W. H., Teukolsky, S. A., Vetterling, W. T. & Flannery, B. P. *Numerical recipes 3rd edition: the art of scientific computing*. 1235 pages (Cambridge University Press, 2007).

15. Liu, Jun S and Liang, Faming and Wong, W. H. The multiple-try method and local optimization in Metropolis sampling. *J. Am. Stat. Assoc.* **95,** 121–134 (2000).
